# Rice sHsp genes: genomic organization and expression profiling under stress and development

**DOI:** 10.1186/1471-2164-10-393

**Published:** 2009-08-24

**Authors:** Neelam K Sarkar, Yeon-Ki Kim, Anil Grover

**Affiliations:** 1 Department of Plant Molecular Biology, University of Delhi South Campus, N Delhi 110021, India; 2 Genomics & Genetics Institute, GreenGene Biotech, Myongji University, 38–2 Namdong Yongin, Kyonggido, Korea

## Abstract

**Background:**

Heat shock proteins (Hsps) constitute an important component in the heat shock response of all living systems. Among the various plant Hsps (i.e. Hsp100, Hsp90, Hsp70 and Hsp20), Hsp20 or small Hsps (sHsps) are expressed in maximal amounts under high temperature stress. The characteristic feature of the sHsps is the presence of α-crystallin domain (ACD) at the C-terminus. sHsps cooperate with Hsp100/Hsp70 and co-chaperones in ATP-dependent manner in preventing aggregation of cellular proteins and in their subsequent refolding. Database search was performed to investigate the sHsp gene family across rice genome sequence followed by comprehensive expression analysis of these genes.

**Results:**

We identified 40 α-crystallin domain containing genes in rice. Phylogenetic analysis showed that 23 out of these 40 genes constitute sHsps. The additional 17 genes containing ACD clustered with Acd proteins of *Arabidopsis*. Detailed scrutiny of 23 sHsp sequences enabled us to categorize these proteins in a revised scheme of classification constituting of 16 cytoplasmic/nuclear, 2 ER, 3 mitochondrial, 1 plastid and 1 peroxisomal genes. In the new classification proposed herein nucleo-cytoplasmic class of sHsps with 9 subfamilies is more complex in rice than in *Arabidopsis*. Strikingly, 17 of 23 rice sHsp genes were noted to be intronless. Expression analysis based on microarray and RT-PCR showed that 19 sHsp genes were upregulated by high temperature stress. Besides heat stress, expression of sHsp genes was up or downregulated by other abiotic and biotic stresses. In addition to stress regulation, various sHsp genes were differentially upregulated at different developmental stages of the rice plant. Majority of sHsp genes were expressed in seed.

**Conclusion:**

We identified twenty three sHsp genes and seventeen Acd genes in rice. Three nucleocytoplasmic sHsp genes were found only in monocots. Analysis of expression profiling of sHsp genes revealed that these genes are differentially expressed under stress and at different stages in the life cycle of rice plant.

## Background

Plants are challenged by fluctuations in environmental factors specially temperature on almost daily basis. High temperature (HT) exerts negative effect on growth and yield of plants [1]. Heat shock response (HSR), defined as sum total of cellular high temperature-related defense activities, is induced upon exposure to HT. Induction of an array of ubiquitous and evolutionary-conserved proteins known as heat shock proteins (Hsps) is one of the main constituents of the HSR [2]. Hsps are divided into high molecular mass proteins comprising of Hsp100, Hsp90, Hsp70/DnaK, Hsp60/GroE and small molecular mass proteins consisting of Hsp20 or small heat shock proteins (sHsps) of 16–42 kDa. sHsps form large oligomeric complexes, ranging in size from 200–800 kDa both in prokaryotic and eukaryotic cells. The characteristic feature of the sHsps is the presence of an evolutionarily-conserved sequence of 80–100 long amino acids called α-crystallin domain (ACD), located in the C-terminal region. The N-terminal region preceding the ACD is variable in length and amino acid sequence and contributes to a large extent towards the structural diversity amongst different sHsps. Hsps generally function as molecular chaperones that facilitate the native folding of proteins in unstressed and stressed conditions and play an important role during stress by preventing irreversible aggregation of denatured proteins. sHsps are shown to be ATP-independent molecular chaperones. Experiments have shown that sHsps form complexes with denatured proteins and prevent their aggregation. From these complexes, the target proteins are subsequently refolded by Hsp100/Hsp70 and co-chaperones in ATP-dependent manner during the recovery phase [3-5]. Expression of Hsps is controlled by heat shock transcription factors (HSFs) that bind to cis-acting regulatory elements called heat shock element (HSEs) in the promoter region of the Hsp genes.

In plants, sHsps are encoded by nuclear multigene families and localized in different cellular compartments. Extensive analysis of *Arabidopsis *sHsp gene family revealed that there are 19 genes coding for sHsps and 25 genes encoding for Acd proteins [6]. In *Arabidopsis*, 13 sHsp genes were categorized into CI, CII and CIII present in cytosol/nucleus, while one each present in chloroplast, endoplasmic reticulum, mitochondrion and peroxisome and 5 sHsp genes were categorized as cytoplasm- related or plastid- related [6,7]. Recently, two groups placed these 5 sHsps into new nucleocytoplasmic and mitochondrial subfamilies that has led to the expansion of nucleocytoplasmic subfamily to 7 subfamilies (I, II, III, IV, V, VI and VII) and mitochondrial subfamily to two subfamilies MI and MII [8,9]. The sHsps gene family is not very complex in bacteria and lower eukaryotes. *E. coli and *S. *cerevisiae *have 2 sHsp each. In human, 10 sHsps have been reported while zebra-fish has 13 sHsps [10]. The possible genes in sHsp family in sugarcane are estimated to be 24 [11]. Higher diversification of plant sHsps may reflect an adaptation to stress conditions that is unique to plants; necessitated because plants being sessile can not escape stress environment and hence may have evolved extended mechanisms to overcome stress. Detailed studies have established that plant sHsps are produced in response to a wide array of environmental insults e.g. heat, cold, drought, high light, UV, osmotic stress, oxidative stress and plant- pathogen interaction [12,13] and their concentration can go up to 1% of the total proteins under heat stress [14]. Some sHsps are highly-expressed in embryogenic tissues and growing fruits [15,16]. Chloroplastic and mitochondrial sHsps are considered to play an important role in heat tolerance [17,18].

Rice is considered a model plant species of the group monocots for its small genome size and availability of large collection of full-length cDNAs (FL-cDNAs) and for the fact that its whole genome is completely sequenced [19]. It is the major food crop being the staple food of about half of the world's population. Spikelet fertility, grain quality and yield processes in rice are challenged by HT [20]. It is anticipated that rise in climate temperature due to global warming would lead to decrease in rice yield even in temperate regions [21]. The grain yield of rice is reported to drop by 10% for every 1°C increase in growing period minimum temperature in the dry season [22]. This indicates that decreased rice yields are associated with increased night time temperature which probably is a result of global warming [22]. To overcome the HT-induced reduction in crop yield, breeding for HT-tolerant crops is crucial [1]. While attempts have been made to understand structural and functional aspects of rice Hsp90 and Hsp100 families [23-27], no major study has been undertaken to delineate the composition and expression analysis of rice sHsp gene family. In rice, CI subfamily, *Hsp18.0 *of CII subfamily and *Hsp26.7-*P have been somewhat characterized previously [28-30]. Over-expression of rice *Hsp16.9-CI *was shown to provide thermo-tolerance to *E. coli *cells [31]. By transgenic approach it was shown that homologous expression of *Hsp17.7-CI *provided heat tolerance, drought tolerance and UV-B resistance [32,33]. To better understand the potential functional relevance of the sHsps in rice, it is important to understand their genomic complexity, expression profiling and tissue distribution.

In this study, genome-wide analysis of rice α-crystallin domain containing genes and their comprehensive expression analysis was performed. To study the expression, analysis of EST database, FL-cDNA and publicly-available microarray data sets for vegetative stages, development and stress treatments was performed. For examining HT stress response of these genes, microarray of rice leaf tissues was carried out. The microarray based expression was supported by RT-PCR analysis of 20 genes. Our results show the complexity and diversity of sHsp gene family of rice at structural and expression level. This is the first report focusing on the comprehensive expression profiles of the ACD containing genes in rice.

## Results

### Complexity and organellar localization of rice sHsp gene family

**Figure 1:**
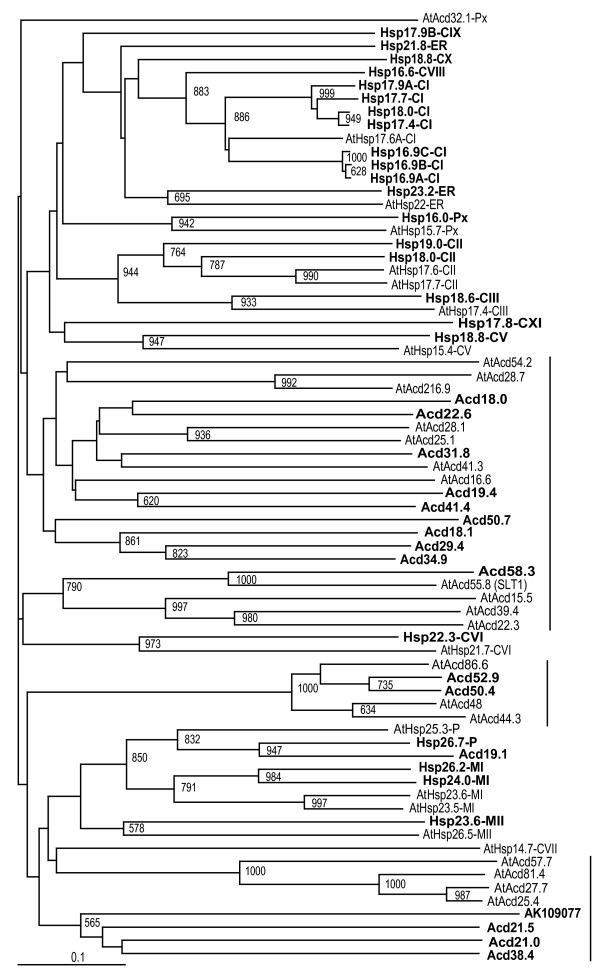
**Phylogenetic analysis of rice ACD gene family**. The tree was derived by Neighbor- joining method with bootstrap analysis (1000 replicates) from alignment of amino acid sequences of conserved ACD of sHsp and Acd of rice and Arabidopsis using CLUSTAL X1.83. The tree was analyzed with TREEVIEW 1.6.6. The bootstrap values >50% are denoted at the nodes. Rice sHsps and Acds are depicted in bold. Clusters of Acd genes are denoted with a vertical line on the right.

**Figure 2:**
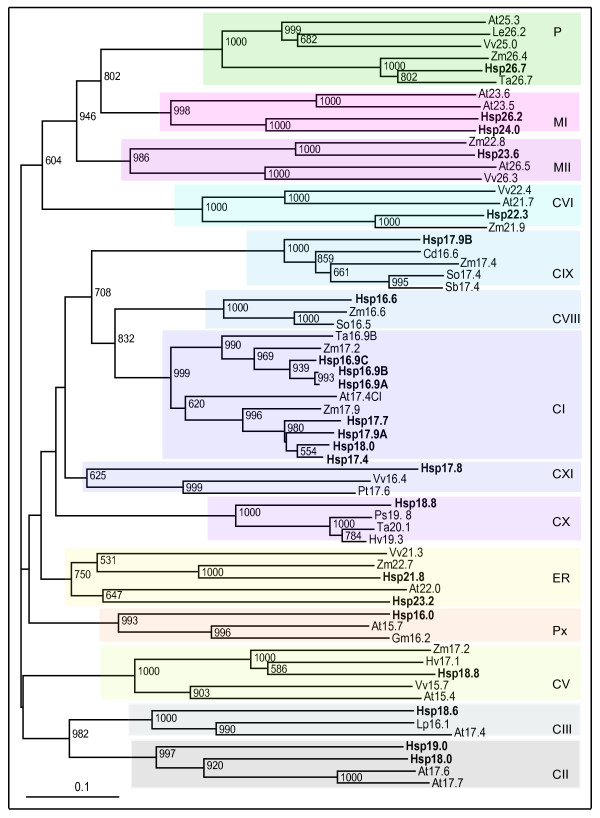
**Phylogenetic relationship of sHsps of rice with diverse plant species**. The tree was generated by Neighbor- Joining method on amino acid sequences of sHsps aligned using Clustal X1.83 and analyzed by TreeView1.6.6. The bootstrap values (>50%) from 1000 replications are indicated at the nodes. The abbreviations of species are as follows: At- Arabidopsis thaliana, Cd- Cynodon dactylon, Gm – Glycine max, Hv- Hordeum vulgare, Lp – Lycopersicon peruvianum, Os- Oryza sativa, Ps- Pseudoroegneria spicata, Pt- Populus tremula, Sb- Sorghum bicolor, So- Saccharum officinarum, Ta -Triticum aestivum, Vv- Vitis vinifera and Zm- Zea mays. Accession number of sHsps are – Cd16.6 (ES295769), Gm16.2 (BAG09378), Hv17.1 (CB878637), Hv19.3 (BF265056), Lp16.1 (AAK84869), Lp26.2 (AAB07023), Ps19.8 (FF349294), Pt17.6 (BU893632), Sb17.4 (CN143862), So16.5 (CA143009), So17.4 (CA184170), Ta16.9B (CAA45902), Ta20.1 (CV766415), Ta26.6 (AAC96315), Vv15.7 (CAO22716), Vv16.4 (CAO64962), Vv21.7 (EE098997), Vv22.4 (CAO39056), Vv25.0 (CAO48583), Vv26.3 (CAO62740), Zm16.6 (ACG25839), Zm17.2 (CAA46641), Zm17.2 (ACG27403), Zm17.4 (ACG45088), Zm17.9 (ACF78669), Zm21.9 (ACG27568), Zm22.7 (ACG31638), Zm22.8 (ACG38401), Zm26.4 (AAA33477). CI – CXI- cytoplasmic/nuclear, ER- endoplasmic reticulum, M- mitochondria, P- plastid, Px- peroxisome. Rice sHsps are shown in bold.

Survey of rice genome at TIGR by keywords alpha crystallin protein, small heat shock protein and heat shock protein was performed to predict genes containing conserved ACD region. Sequences obtained by keyword search were used as query for BLAST in NCBI database and the output sequences were screened for the presence of ACD in PROSITE. After removing overlapping sequences, 40 sequences containing ACD in rice genome were retrieved. Details of all the genes encoded by these sequences are presented in Table 1. Phylogenetic tree constructed using these 40 sequences and various sHsps and Acd proteins of *Arabidopsis *revealed that 23 rice genes grouped with sHsps and 17 genes with regions sharing homology to ACD, diverged from sHsps and grouped with Acd proteins from *Arabidopsis *(Figure 1). We however, noticed two exceptions in this clustering pattern: (1) *Hsp22.3*-*CVI *along with recently categorized *AtHsp21.7-CVI *[8] was present on the clade with Acd genes and (2) Acd19.1 of rice was clustered with sHsps, showing their proximity to respective genes. Furthermore, 5 sHsp genes of rice (*Hsp16.6-CVIII*, *Hsp17.9B-CIX*, *Hsp18.8-CX*, *Hsp17.8-CXI *and *Hsp21.8-ER*) were not clustered with any of the sHsp genes of Arabidopsis. A search in the EST database of other plant species was further performed to check for homologs of these genes. Subsequently, phylogenetic tree generated by alignment of amino acid sequence of rice sHsps and ESTs (Additional file 1) showed that these 5 sHsp genes grouped into distinct clusters on separate clades with sHsps from other plant species, reflecting that homologs of above mentioned genes were indeed present in other plants (Figure 2). Based on the phylogenetic tree and *in silico *localization analysis (Figure 2 and Additional file 2), we identified rice sHsp members relating to previously defined subfamilies CI, CII, CIII, M, P, ER and Px as well as recently identified subfamilies CV, CVI and MII [7-9]. In addition, we identified four additional nucleocytoplasmic subfamilies in rice each represented by one sHsp gene. These new subfamilies are CVIII (*Hsp16.6*), CIX (*Hsp17.9B*), CX (*Hsp18.8*) and CXI (*Hsp17.8*) (Figure 2). Considering that there are 7 nucleocytoplasmic subfamilies in *Arabidopsis *we have numbered the newly identified subfamilies from VIII onwards. Homologous genes for *Hsp16.6-CVIII, Hsp17.9B-CIX *and *Hsp18.8-CX *were found only in monocots and *Hsp17.8-CXI *subfamily was found to have homologous genes in dicots. Thus, the 23 sHsps of rice consist of 14 subfamilies distributed to various cellular organelles in the following manner: 16 nucleo-cytoplasmic (C) sHsps (9 subfamilies), 3 mitochondrial (M) sHsps (2 subfamilies), 2 endoplasmic reticulum (ER)-localized sHsps, 1 plastidial (P) sHsp and 1 peroxisomal (Px) sHsp having 1 subfamily each. As against our analysis, 22 genes are annotated as sHsps and 8 genes as sHsp-like or ACD proteins in TIGR rice genome annotation database. 22 sHsps genes in TIGR are further annotated as 12 CI sHsps, 3 CII sHsps, 3 chloroplastic sHsps and 2 each as ER and mitochondrial.

**Table 1:** Features of sHsps and Acds genes of rice

**Protein name**	**Gene ID** **(TIGR)**	**Chromosome location and orientation**	Intron*	**FL-cDNA**
**sHsps**				
Hsp16.6-CVIII	Os01g04340	1: (+) 1933247–1933699	NI	AK063681
Hsp17.9B-CIX	Os01g04350	1: (+) 1940149–1940649	NI	AK119599, AK064849, AK065690, AK062091
Hsp16.9C-CI	Os01g04360	1: (-) 1943473–1943922	NI	n/a
Hsp16.9A-CI	Os01g04370	1: (-) 1948005–1948457	NI	n/a
Hsp16.9B-CI	Os01g04380	1: (+) 1951047–1951499	NI	AK121025
Hsp18.0-CII	Os01g08860	1: (-) 4448290–4448790	NI	AK071240, DQ180746
Hsp18.8-CX	Os02g03570	2: (+) 1450233–1450766	NI	n/a
Hsp23.6-MII	Os02g10710	2: (-) 5639315–5640360	SI [97(386n)122]	AK106682
Hsp19.0-CII	Os02g12610	2: (-) 6616538–6617065	NI	CT835445
Hsp17.8-CXI	Os02g48140.1 Os02g48140.2	2: (+) 30355328–30355822	NI	CI140562 AK107963
Hsp24.0-MI	Os02g52150	2: (+) 32807782–32808549	SI [83(105n)137]	AK105464, AK074003, AK064389
Hsp18.6-CIII	Os02g54140	2: (+) 34066403–34067009	SI [82(87n)90]	AK119261, AK063602
Hsp26.7-P	Os03g14180	3: (+) 7746457–7747179	NI	AK063618, AK120045, AK120048, AB020973
Hsp17.9A-CI	Os03g15960	3: (+) 8856447–8856932	NI	AK104129, AK119616, AK119664, AK073671, AK119239, AK119675
Hsp17.4-CI	Os03g16020	3: (-) 8884576–8885040	NI	AK119243, AK119717
Hsp18.0-CI	Os03g16030	3: (+) 8885608–8886093	NI	n/a
Hsp17.7-CI	Os03g16040	3: (+) 8888694–8889173	NI	AK069547
Hsp23.2-ER	Os04g36750	4: (+) 22300635–22301282	NI	AK063700
Hsp22.3-CVI	Os05g42120	5: (+) 24674434–24675920	SI [111(875n)92]	AK110627
Hsp26.2-MI	Os06g11610	6: (-) 6151339–6152222	SI [103(137n)145]	n/a
Hsp16.0-Px	Os06g14240	6: (-) 7940169- 7940609	NI	AK105317
Hsp18.8-CV	Os07g33350.1 Os07g33350.2	7: (+) 20595735–20596341	SI [73(85n)100]	AK099296 AK063798
Hsp21.8-ER	Os11g13980	11: (-) 7752380–7753000	NI	AK107883
**Acds**				
Acd18.1	Os01g40530	1: (-) 24554327–24555689	SI	n/a
Acd29.4	Os01g40550	1: (-) 24558012–24558960	SI	AK109917
Acd58.3	Os01g62300	1: (-) 37800329–37801894	NI	AK069954, AK103961
Acd52.9	Os02g48370	2: (-) 30498592–30502991	MI	AK107036
Acd19.1	Os03g61940	3: (-) 35964494–35965033	NI	n/a
Acd41.4	Os03g45330	3: (-) 26352936–26356577	SI	n/a
Acd31.8	Os03g45340	3: (-) 26360084–26361059	SI	AK062338
Acd22.6	Os03g06170	3: (-) 3074366–3075216	SI	n/a
Acd34.9	Os05g51440	5: (+) 29576192–29577284	SI	n/a
Acd50.4	Os06g41730	6: (+) 25891207–25895963	MI	AK073058, AK099075, AK101550, AK064549
Acd21.5	Os09g17660	9: (+) 11366789–11367960	SI	n/a
Acd38.4	Os10g07200	10: (-) 3758835–3760441	SI	AK064267
Acd21.0	Os10g07210	10: (-) 3764967–3766298	SI	AK107162
Acd18.0	Os10g30162	10: (+) 16138850–16140552	SI	AK062774
Acd19.4	Os10g30180	10: (+) 16145983–16147060	SI	n/a
Acd50.7	Os12g06820	12: (-) 3316867–3318361	SI	AK110577
Acd30.2	n/a	n/a		AK109077

n/a – not available, NI – no intron, SI – single intron, MI – multiple introns, C – cytoplasm/nuclear, M – mitochondria, ER – endoplasmic reticulum, P – chloroplast, Px – peroxisome. * The number in bracket shows the intron length in nucleotides. Numbers preceding and following the bracket represent exon1 and exon2 (in amino acids), respectively.

Among the 17 Acd genes in rice we identified one FL-cDNA in KOME database (AK109077), whose conceptual translation displayed that it has 2 ACDs arranged in tandem. Position of this full length clone in the genome could not be mapped on chromosome. Two genes, *Acd52.9 *and *Acd50.5 *were found to have ARID or bright domain in addition to the ACD. The ARID containing proteins from rice as well as *Arabidopsis *were found to be chromatin-associated proteins  and are suggested to be involved in chromatin remodeling. Several Acd proteins were found to have predicted transmembrane domain. Intracellular localization of Acd19.1 (Os03g61940) is predicted to be ER in Predotar and Acd38.4 (Os10g07200), Acd21 (Os10g07210) and Acd21.5 (Os09g17660) show plastid localization. Nonetheless, these protein sequences do not show any consensus sequences which is present in ER localized proteins or chloroplastic sHsps.

Sequence alignment of amino acids of sHsps showed that members of different subfamilies do not share high sequence similarity, yet the secondary structure is conserved across subfamilies. The 'GVL' residues which are highly conserved in the consensus region I are variable in Hsp17.9B-CIX and in Hsp22.3-CVI subfamily (Additional file 3). The β6 strand was noted to be absent in CV sHsps of dicots. The β10 strand which has conserved sequence motif, basic-x-I/V-x-I/V, was absent in Hsp22.3-CVI subfamily. It is proposed that the amino acids from β10 strand facilitate inter-subunit linkage for oligomerization of sHsps [34].

**Figure 3:**
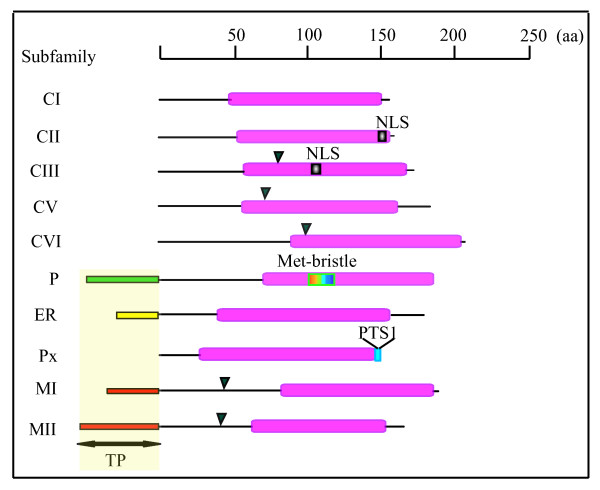
**Diagramatic illustration of sHsp subfamilies of rice**. The conserved α- crystallin domain of sHsps is shown in pink. The transit peptide (TP) of the organellar sHsps, the NLS of CII and CIII sHsp, the methionine rich region (Met-bristle) of plastidial sHsp and peroxisomal targeting signal (PTS1; SKL) are marked. The position of the introns is indicated with black arrowhead. Description of sHsp subfamilies is given in the text. Intron- exon structure of subfamilies CVIII, CIX, CX and CXI is similar to CI subfamily.

Twenty three sHsps and 16 Acd mapped genes (1 Acd gene could not be mapped as stated above) are distributed over all chromosomes, except on chromosome 8. Interestingly, closely related sequences of CI subfamily that clustered together in the phylogenetic tree are present on chromosome 1 and chromosome 3 suggesting that expansion of this gene family may have occurred due to localized or intra-chromosomal duplication. In addition, one sHsp and two Acd genes showed segmental duplication: these include genes present on duplicated chromosomal segments between chromosome 2 and 6 (*Hsp24-MI *and *Hsp26.2-MI*), chromosome 3 and 10 (*Acd22.6 *and *Acd18*) and chromosome 1 and 5 (*Acd29.4 *and *Acd34.9*). Alignment of available FL-cDNA and ESTs sequences with respective genomic DNA revealed that 6 sHsp genes are interrupted by one intron each and 17 sHsp genes have no intron. On the contrary, the Acd genes were interrupted by either single or multiple introns (Table 1). The diagrammatic representation of sHsp subfamilies showing ACD, intron position and transit peptide is illustrated in Figure 3.

### Expression analysis of rice sHsp and Acd genes

The levels of expression of sHsp and Acd genes of rice were investigated by two approaches. The first approach was based on scrutiny of digital expression profile of ESTs (TIGR, gene expression evidence), FL-cDNA (KOME database) and microarray data available at Genevestigator [35]. The latter source facilitated the analysis of the whole genome microarray based expression data for vegetative stage, developmental stages as well as various environmental stresses excluding heat stress. In the second approach, heat stress induced expression analysis of sHsps and Acds genes was performed by analyzing whole genome microarray data of leaf tissues of rice (Sarkar et al, unpublished data,). Expression of sHsps was further analyzed by RT-PCR analysis.

**Figure 4:**
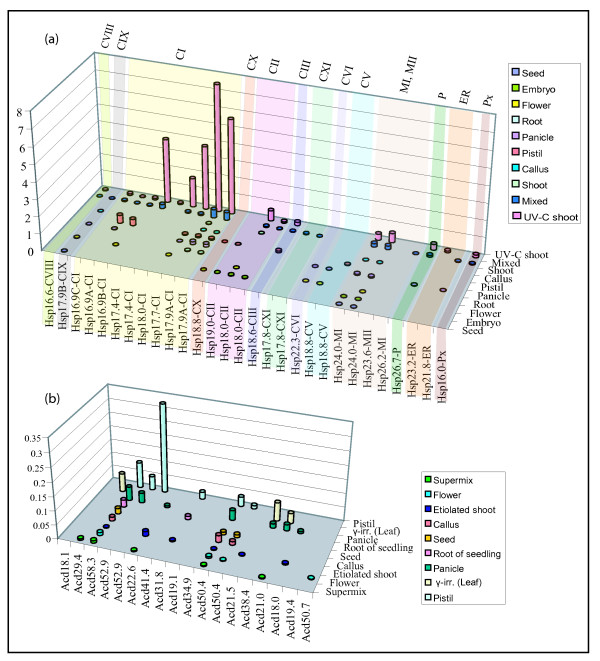
**EST analysis of sHsp and Acd genes of rice**. EST based expression of sHsp genes (a) and Acd genes (b). The details of the libraries are given in 'methods'. The library for each tissue is not same for each gene. Expression of sHsps and Acd genes is plotted as percent frequency i.e. (number of ESTs for particular gene/total number of ESTs in the library). γ-irr; gamma irradiated.

The digital expression profiles derived from the abundance of ESTs in diverse libraries (nine non-stresses and one UV-C stress library) for *sHsps *and *Acds *showed that all sHsp genes except *Hsp23.6-MII *were expressed in various libraries (Figure 4). Highest level of ESTs were observed in UV-C stress library for CI sHsps and a distinct induction in the ESTs was noticed for CII, CIII, MI, P and Px sHsps (Figure 4). ESTs for newly identified subfamilies *Hsp17.9B-CIX*, *Hsp18.8-CX*, *Hsp17.8-CXI*, *Hsp18.8-CV *and *Hsp22.3-CVI *were absent in UV-C stress library. Expression of most of the *sHsp *genes (except *Hsp19.0-CII*, *Hsp18.8-CX*, *Hsp23.5-MII *and *Hsp18.8-CV*) was found in mixed-library though the EST abundance was much lower as compared to that in stress library. Expression of *Hsp18.8-CV *was noted in flower and panicle while *Hsp19.0-CII *was restricted to the embryo and pistil libraries suggesting that the expression of these sHsps may be regulated in specific tissues or at developmental stages. Expression of alternative splice variants of six sHsp genes was observed in EST analysis (Figure 4). However, we noted FL-cDNA and EST support for alternative spliced variant of *Hsp18.8-CV *and *Hsp17.8-CXI*. (Table 1). No expression evidence was found for one sHsp gene and 5 Acd genes in EST analysis. Expression of four sHsps and seven Acd genes was not supported by FL-cDNAs (Table 1).

#### Developmental stage specific expression of sHsps

**Figure 5:**
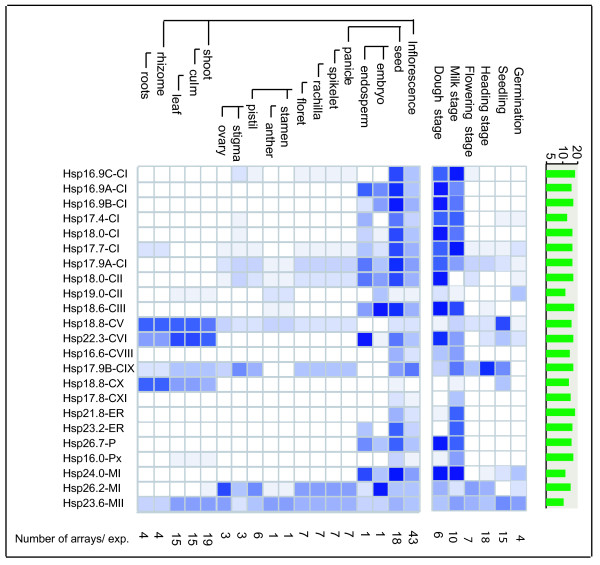
**Microarray based expression profiles of sHsp genes during developmental stages of rice plant**. Expression of sHsp genes during developmental stages are presented as heat maps in blue/white colors generated using meta-analysis tool at Genevestigator . The darker color corresponds to stronger expression.

Microarray based expression analysis of developmental stages of rice plant showed that transcripts of most of the sHsp genes were widely expressed although their expression levels varied at different developmental stages (Figure 5). *Hsp17.9B-CIX, Hsp18.8-CX*, *Hsp23.6-MII, Hsp18.8-CV *and *Hsp22.3-CVI *were expressed constitutively in both root and shoot. The latter two genes were expressed in shoot, leaf and culm in moderately high levels. In stigma and ovary, transcripts of seven sHsp genes (*Hsp17.7-CI*, *Hsp17.9A-CI*, *Hsp18.0-CII*, *Hsp18.8-CV*, *Hsp17.9B-CIX*, *Hsp26.2-MI *and *Hsp23.6-MII*) were present in moderate level. In various parts of panicle, eight sHsp genes (*Hsp16.9C-CI*, *Hsp17.9A-CI*, *Hsp17.9B-CIX*, *Hsp17.7-CI*, *Hsp18.0-CII*, *Hsp18.8-CV*, *Hsp26.2-MI and Hsp23.6-MII*) displayed varied expression. We further noted that transcripts of CI sHsp genes located on chromosome 3 (*Hsp17.4, Hsp17.7, Hsp17.9A *and *Hsp18.0*), *Hsp26.2-MI *and *Hsp19.0-CII *were present in high abundance during early stages of development of pollen and tapetum as shown by laser micro dissection microarray data analysis of anther development [36] (Additional File 4). In contrast, during late stages of anther development (from uninuclear microspore to tricellular pollen) significant expression of *Hsp17.9A *and *Hsp19.0-CII *was observed. During various stages of seed development, sHsp genes from most of the subfamilies exhibited differential expression.

#### Stress induced expression of sHsps

**Figure 6:**
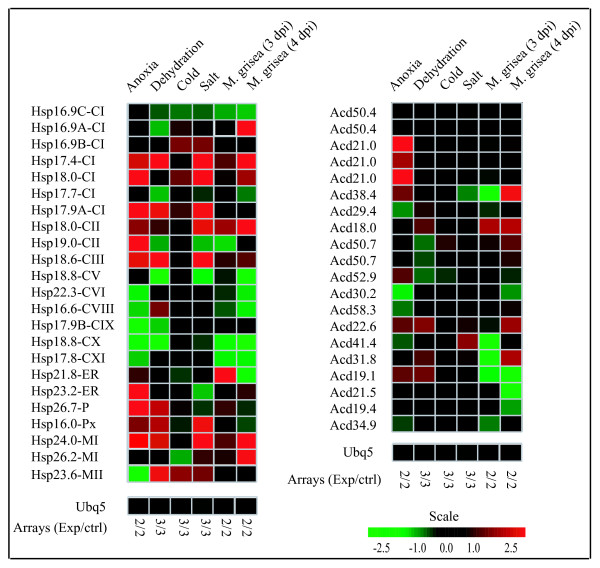
**Expression pattern of sHsp and Acd genes under various stress conditions**. The microarray data based expression profiles under stress conditions are presented as heat maps generated using meta-analysis tool at Genevestigator . The transcript levels are depicted by color scale indicating log_2 _values. Ubq5 expression is shown as control.

Microarray based analysis of 7 d old seedlings of rice under LT stress (3 h, 4°C) revealed that except for *Hsp16.9C-CI*, *Hsp18.0-CI *and *Hsp23.6-MII *which are upregulated, expression of most of the *sHsp *genes is unaltered (Figure 6). Salt stress (3 h, 200 mM NaCl) caused upregulation of ten genes and down regulation of three genes. Under dehydration stress enhanced transcript level of eight sHsp genes and reduced transcript of six genes was observed. Five genes (*Hsp17.9A-CI, Hsp17.4CI, Hsp18.6-CIII, Hsp24.0-MI *and *Hsp16.0-Px*) showing upregulation were common in salt and drought stress. Transcript level of most of the sHsp genes was responsive to anoxia stress. Anoxia stress caused drastic enhancement of the transcript level (>10 fold) of *Hsp17.9A-CI*, *Hsp18.0-CI *and *Hsp24.0-MI *whereas transcript of *Hsp17.4-Cl*, *Hsp18.0-CII*, *Hsp19.0-CII*, *Hsp18.6-CIII*, chloroplast and ER sHsps were moderately up-regulated [37]. However, six genes (*Hsp22.3-CVI*, *Hsp16.6-CVIII*, *Hsp17.9B-CIX*, *Hsp18.8-CX, Hsp17.8-CXI *and *Hsp23.6-MII*) were down-regulated in anoxia (Figure 6). In response to sodium arsenate stress in roots of rice, nine genes (*Hsp16.9A-CI*, *Hsp16.9B-CI*, *Hsp16.9C-CI*, *Hsp17.4-CI, Hsp18.0-CI*, *Hsp18.6-CIII*, *Hsp26.7-P*, *Hsp26.2-MI *and *Hsp21.8-ER*) were shown to be up-regulated [38]. In response to biotic stress [*M. grisea*, 4 days post infection (dpi)], *Hsp17.4-CI, Hsp18.0-CI, Hsp16.9A-CI, Hsp18.0-CII *and both MI genes were upregulated and 4 genes (*Hsp22.3-CVI*, *Hsp16.6-CVIII*, *Hsp18.8-CX *and *Hsp17.8-CXI*) were down regulated.

**Figure 7:**
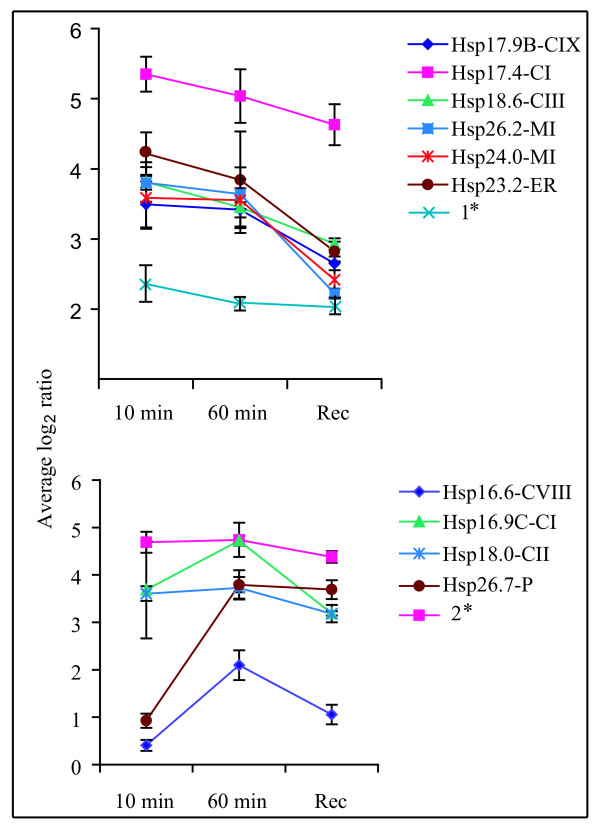
**Microarray analysis of expression profiles of sHsps of rice under heat stress**. Leaves from one month old plants were given stress at 42°C. Samples were harvested after 10 min and 1 h of HT treatment and 30 min recovery following 1 h HT. Three replicates were performed and the standard error is shown by error bars. 1* represents a probe which cross-reacts with *Hsp17.9A-CI*, *Hsp18.0-CI*, *Hsp17.4-CI *and *Hsp17.7-CI*. 2* represents a probe on the microarray chip which overlaps with *Hsp16.9A-CI*, *Hsp16.9B-CI*, *Hsp16.9C-CI*, *Hsp17.9A-CI*, *Hsp18.0-CI *and *Hsp17.4-CI*.

To get insight into the expression profiling of the sHsp gene family under heat stress, microarray data of rice leaf tissues was analyzed. The probes for *Hsp21.8-ER *and *Hsp23.6-MII *were absent on the 60K chip used for expression analysis. In addition, there are two probes (Os046986_01 and Os033333_01) on the chip showing cross-reactivity with seven CI sHsps (*Hsp17.9A-CI*, *Hsp18.0-CI*, *Hsp17.4-CI, Hsp17.7-CI, Hsp16.9A-CI*, *Hsp16.9B-CI *and *Hsp16.9C-CI*). Hence, there were no specific probes for *Hsp17.9A-CI, Hsp18.0-CI, Hsp16.9A-CI *and *Hsp16.9B-CI*. The expression of sHsp genes belonging to various subfamilies was highly up-regulated under HT (Figure 7). Transcript level of 10 sHsp genes showed up-regulation by more than 5 fold within 10 min at 42°C. Overall, expression of sHsps under HT followed 3 types of kinetics: Type I where expression was initially enhanced after 10 min at 42°C, on further exposure for 1 h decrease in the transcript level was registered (represented by *Hsp17.4-CI*, *Hsp17.9B-CIX*, *Hsp23.2-ER*, *Hsp18.6-CIII*, *Hsp24.0-MI*, *Hsp26.2-MI*); Type II where up-regulation within 10 min at 42°C was further enhanced upon increasing the stress duration to 1 h (represented by *Hsp16.6-CVIII*, *Hsp16.9C-CI *and *Hsp18.0-CII*); Type III where there was no significant change in the expression of sHsp genes till 10 min at 42°C but the transcript level was upregulated drastically (more than 8 folds) after 1 h at 42°C (represented by *Hsp26.7-P*). No change in the transcript level of *Hsp16.0-Px, Hsp22.3-CVI, Hsp18.8-CV, Hsp17.8-CXI *and *Hsp18.8-CX *was observed. Transcripts of all the upregulated sHsp genes were retained after 30 min of recovery, albeit a marginal decline in the level was observed.

**Figure 8:**
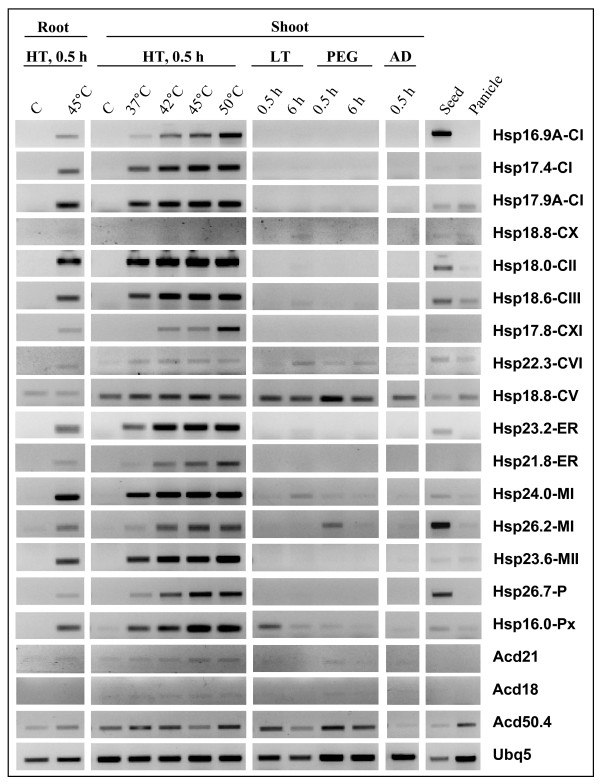
**Semi-quantitative RT-PCR analysis of sHsps expression under heat, cold, air drying and osmotic stress**. Total RNA from the roots and shoots of 7 d old seedlings, dry seeds and panicle was used for RT-PCR analysis. Heat stress (HT): 0.5 h at various temperatures; Osmotic stress: 15% PEG 4000; Dehydration stress: air drying (AD) 20 min; low temperature (LT): 6°C ± 2. Ubq5 was used as internal control for RT reaction.

Subsequent expression analysis of *sHsp *genes by semi-quantitative RT-PCR using RNA from root and shoot validated that transcript of most of the *sHsp *genes were upregulated in both the tissues under HT. The transcripts of *Hsp18.8-CV *and *Hsp22.3-CVI *were constitutively present and their level was unaltered under heat, cold, dehydration and salt stress (Figure 8). Constitutive expression of *Hsp18.8-CV *and *Hsp22.3-CVI *and low transcript abundance of *Hsp22.3-CVI *in comparison to *Hsp18.8-CV *is commensurate with the expression data from published microarrays (Figure 5). At 37°C for 30 min, significant levels of transcript were noted for eight genes (*Hsp17.4-CI*, *Hsp17.9A-CI*, *Hsp18.0-CII*, *Hsp18.6-CIII*, *Hsp23.2-ER*, *Hsp24.0-MI*, *Hsp23.6-MII *and *Hsp16.0-Px*) whereas marginal increase in the transcript level of 5 genes (*Hsp16.9A-CI*, *Hsp21.8-ER*, *Hsp17.8-CXI*, *Hsp26.2-MI *and *Hsp26.7-P*) was observed. At temperatures higher than 37°C, further increase in the transcript level of sHsps was observed. We noted that the expression behavior of two sHsp genes in semi-quantitative RT-PCR is not in agreement with the results of microarray. *Hsp16.0-Px *and *Hsp17.8-CXI *did not show HT inducibility in microarray; however, transcript level of these two genes was upregulated under heat stress conditions in RT-PCR. It was further noted that expression of *Hsp24.0-MI *and *Hsp18.6-CIII *was in cold and dehydration stress (Figure 8). In panicle, feeble signal for expression of *Hsp17.9A*-*CI*, *Hsp18.6-CIII, Hsp18.8-CV *and *Hsp22.3-CVI *was noticed. In mature seed, expression pattern of sHsp genes was concurrent with the microarray based expression. No expression was seen for *Hsp18.8-CX, Hsp19.0-CII *and *Acd38*.4 under the stress condition used for RT-PCR, though the expression of latter two genes is supported by FL-cDNA clones (Table 1).

#### Expression analysis of Acd genes

Acd genes were also widely expressed in various developmental stages. Unlike sHsp genes, most of the Acd genes were found to be constitutively expressed in root and shoot. *Acd50.4*, *Acd58.3*, *Acd19.1*, *Acd34.9 *and *Acd19.4 *were expressed during developmental stages of rice plant (Additional file 5). In general, expression of Acd genes was not affected significantly by cold, salt, dehydration and anoxia stress except *Acd21.0 *which showed upregulation under anoxia stress (Figure 6). Expression of Acd genes was either unaffected or downregulated under heat stress (Additional file 5). However, two Acd genes, *Acd21.0 *and *Acd30.2 *showed upregulation under HT. RT-PCR analysis showed that transcripts of *Acd21.0*, *Acd18.0 *and *Acd50.4 *genes displayed constitutive expression and remained unaffected under stress conditions.

### *In silico *analysis of promoter regions of sHsp genes

**Figure 9:**
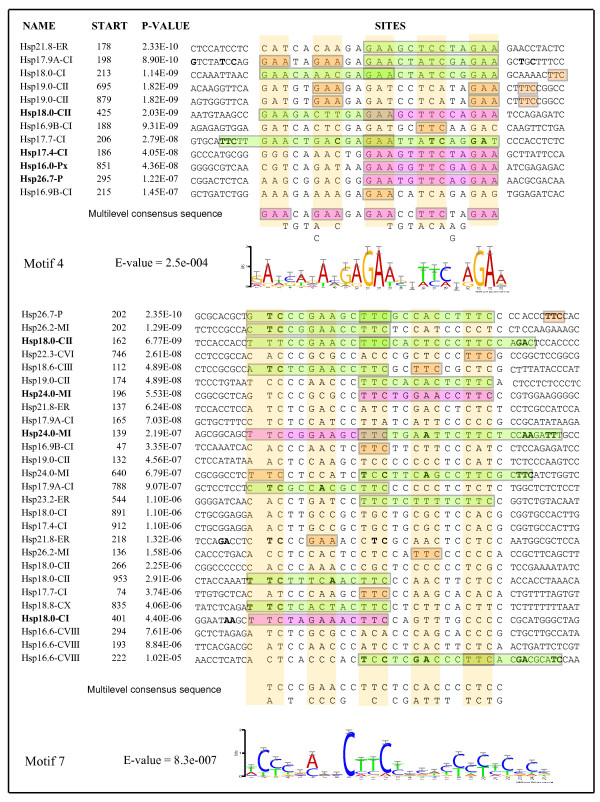
**MEME based consensus sequences in the promoters of sHsp**. The motifs obtained by MEME analysis were plotted according to their positions within the sites and their consensus sequences were presented as graphs using MEME LOGO. The occurrences of motif is sorted by P-value and aligned with each other. The E-value of motif is an estimate of the expected number of motifs with the same width and number of occurrences that would be present in a similarly sized set of random sequences. The height of symbols in each stack in the motif indicates the sequence conservation at that position. The sequences are manually highlighted to show recognized consensus HSEs: pink box-nGAAnnTTCnnGAAn or nTTCnnGAAnnTTCn showing perfect HSE, green box-showing imperfect module of HSE. All nGAAn and nTTCn are shown in orange boxes. The 'Start' indicates the distance from translational start site (ATG). Nucleotides in bold indicate the consensus bases present in HSEs.

Expression of Hsp genes is regulated at transcriptional level by binding of HSFs to HSEs. The analysis of promoter region of sHsp genes (1.2 kb upstream of ATG), using PLACE database showed that HSEs were present only in the promoters of *Hsp17.4-CI *and *Hsp18.0-CI *genes. Further investigation showed that this database recognizes 5'-CTnGAAnnTTCnAG-3' as HSE module. As varied combinations of palindromic repeats of nGAAn are considered functional HSEs [39], we subsequently followed manual inspection and motif-based sequence analysis tool MEME (multiple Em for motif elicitation) for motif search. For the latter approach we opted to select 7 motifs (instead of default setting of 3 motifs). Out of these 7 different motifs (Additional file 6), 2 relevant motifs (#4 and #7, Figure 9) showed consensus sequences similar to known palindromic nGAAn (perfect) and its variants (imperfect) in many sHsp genes. From these analyses, it emerged that promoters of seven sHsp genes (*Hsp18.0-CI*, *Hsp17.4-CI*, *Hsp17.4-CI*, *Hsp18.0-CII, Hsp26.7-P*, *Hsp24.0-MI *and *Hsp16.0-Px*) contained a perfect HSE module (nGAAnnTTCnnGAAn or nTTCnnGAAnnTTCn or both). Promoter regions of other sHsps showed imperfect HSE module (Figure 9 and additional file 7). Majority of the HSEs were located proximal to the ATG. Distal HSEs were noticed in the promoters of *Hsp16.0-Px*, *Hsp17.7-CI *and *Hsp23.6-MII*. None of the promoters of Acd genes were found to have perfect HSEs. However, nnGAAnnTTC or nnTTCnnGAAnn was present in *Acd19.1*, *Acd21.5 *and *Acd18.0 *promoters (results not shown).

## Discussion

Based on genome-wide analysis of ACD containing gene family, this study shows that rice has 23 sHsp and 17 Acd genes. In contrast, *Arabidopsis *has 19 sHsps and 25 Acd proteins [6]. In earlier attempts on categorization of *Arabidopsis *sHsps, it was proposed that while 5 genes do not fall in distinct subfamilies, rest of the sHsps can be placed into 7 subfamilies (namely CI, CII, CIII, M, P, ER and Px) [6,7]. In a more recent attempt, *Arabidopsis *sHsp gene family has been extended to 12 subfamilies by placing the 5 uncategorized sHsp genes into 4 new nucleocytoplasmic subfamilies [namely CIV (*AtHsp18.5*), CV (*AtHsp15.4*), CVI (*AtHsp21.7*) and CVII (*AtHsp14.7*)] and by adding a new mitochondrial subfamily MII (*AtHsp26.5*) [8]. The latter study further showed that homologous genes of *Arabidopsis *CIV and CVII subfamily are not present in rice [8]. Waters et al [9] performed comparative analysis of sHsp gene family of *Arabidopsis*, *Populus *and rice and suggested that the plant sHsp gene family may be categorized into 11 subfamilies. This group indicated that 5 sHsp genes of rice namely *Os16.9C *(Os02g48140), *Os17.6A *(Os01g04340), *Os18 *(Os11g13980), *Os18.2 *(Os02g03570) and *Os21.2 *(Os02g10710) are so-called orphan genes because their homologs are not found in *Populus *and *Arabidopsis*. Our analysis shows that gene entry corresponding to Os02g10710 (*Hsp23.6-MII *in this study) has a definite homolog in *Arabidopsis *(Figure 1). We further find that *Hsp17.9B-CIX *(Os01g04350) gene also does not have homologous counterpart in *Arabidopsis*. We show that homologs of rice entries corresponding to Os01g04340 (*Hsp16.6-CVIII *in this study), Os01g04350 (*Hsp17.9B-CIX*) and Os02g03570 (*Hsp18.8-CX*) are actually present in monocots, homologs of Os02g48140 (*Hsp17.8-CXI*) are present in dicots and homologs of Os11g13980 (*Hsp21.8-ER*) are present in both monocots and dicots. These genes in bootstrap NJ tree analysis segregated into separate clades which are supported by significant bootstrap score of 70% and above. Based on comprehensive analysis done in this work, we propose that sHsp gene family of rice is constituted of 14 subfamilies. We show that there are nine nucleocytoplasmic subfamilies in rice and CI subfamily is the largest with seven members.

This study highlights that 74% of rice sHsp genes are intronless based on genomic organization data. In rice, overall ~20% of the genes are intronless [40]. Importantly, the length of the introns in intron harboring sHsp genes of rice is relatively short (Table 1). Intron length is reported to be inversely proportional to gene expression levels in humans and worms and positively associated with expression level in plants [41,42]. There are indications that introns pose hindrance to rapid gene regulation and are selected against in those genes which require quick adjustment in transcript level to overcome the environmental challenges [43]. Incidentally, sHsp genes are one of the rapidly expressed genes as sHsp transcripts are observed within 10 min of HT in most cases. The absence of introns or their presence with smaller size may thus have correlation to rapid induction needs of sHsp genes. Further, orthologous genes generally tend to maintain the same exon-intron structure [44]. We observed an interesting point regarding intron in plastidial sHsp genes: the plastidial sHsp gene of *Arabidopsis *has one intron while the intron is absent in plastidial sHsp gene of rice. On further examination, we note that plastidial sHsp genes in dicots like *Populus, Vitis *and *G. arboreum *also contain one intron. Intron was noted to be present in chloroplast sHsp gene of monocots like maize and bentgrass (*Agrostis stolonifera var. palustris*) but absent in rice. Wild rice *O. minuta *also has intronless chloroplastic sHsp gene. This may thus suggest that intronloss feature in chloroplastic sHsp gene of rice may have appeared after their divergence from common ancestor into subfamily Erhartoideae (to which rice belongs) and Panicoideae (to which maize belongs).

**Figure 10:**
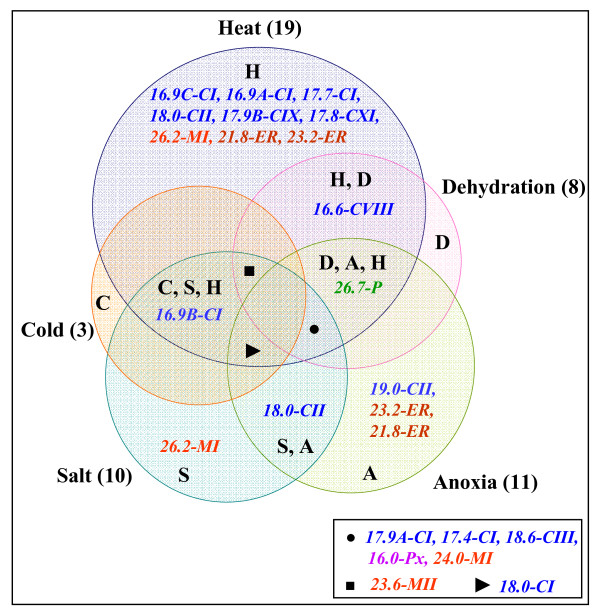
**Overlapping expression of sHsp genes in various abiotic stresses**. The numbers in brackets denote the total number of sHsp genes that are upregulated under each stress treatment (H-heat, C- cold, S- salt, A-anoxia and D- dehydration stress). Black circle denotes overlapping expressed genes in H, D, A and S stresses, black triangle denotes genes expressed in H, C, A and S stresses and black square denotes genes expressed in H, C, D and S stresses. Nucleo-cytoplasmic sHsp members are shown in blue font, mitochondrial sHsps are shown in red font, ER sHsps are shown in brown font, peroxisomal sHsps are shown in purple font and chloroplastic sHsps are shown in green font.

**Figure 11:**
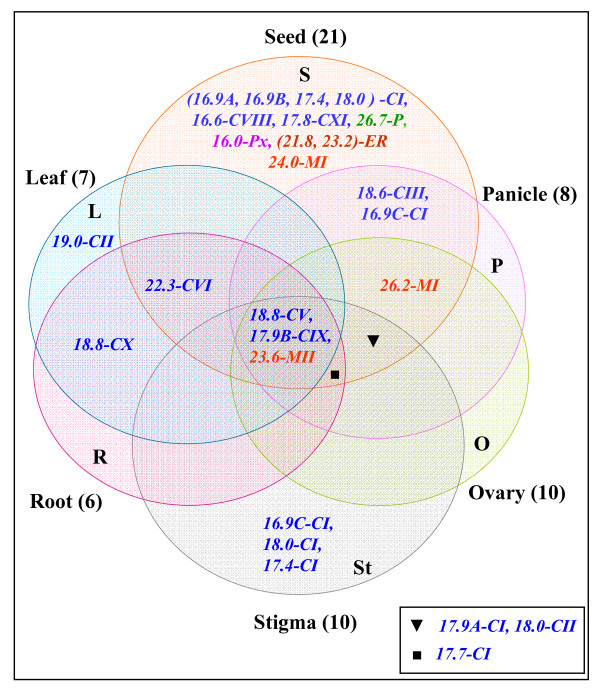
**Overlapping expressions of sHsps in different developmental stages of rice**. The color code of the fonts is same as shown in legend of Figure 10.

From the expression analysis, it is evident that there is spatio-temporal regulation of rice sHsp genes under stress and developmental stages. An overview of sHsp expression under stress and development is presented in Figure 10 and Figure 11. It is evident from this presentation that the expression of sHsp genes is mainly associated with heat stress (and other stresses) as well as under unstressed conditions in vegetative tissues, pollens and seeds. Various nucleocytoplasmic sHsps [sHsps of CI subfamily, one of the CII sHsp (*Hsp18.0*) and CIII sHsp] followed this typical expression pattern under HT and seed development. In an earlier study, sHsps of CI were shown to be present in dry seeds of rice [29]. In pollens, all CI sHsps were not expressed. This observation is in agreement with the CI sHsp expression in *Arabidopsis*. *Hsp19.0-CII *gene was not induced by HT and its transcript was barely detectable in seed. In *Arabidopsis*, both the CII members were shown to be induced by HT. Expression profile of CV and CVI sHsps differed from CI, CII and CIII sHsps. These genes were expressed constitutively in root, shoot and leaf. Moreover, expression of genes from both CV and CVI subfamilies was not altered by HT. The homologous genes of rice *Hsp18.8-CV *and *Hsp22.3-CVI *in *Arabidopsis *(namely *Hsp15.4-CV *and *Hsp21.7-CVI*, respectively) also are expressed constitutively in vegetative tissues [8]. While expression of *AtHsp21.7-CVI *was unaffected by HT, expression of *AtHsp15.4-CV *was rather downregulated. Expression behavior of CV and CVI sHsp genes differed during developmental stages as well. *Hsp18.8-CV *was expressed in all parts of panicle and showed feeble expression in seed, and *Hsp22.3-CVI *was moderately expressed in seed only. In comparison, the homologous genes of *Arabidopsis *were not expressed in seed. Amongst the monocot specific sHsps, two genes (*Hsp17.9B-CIX *and *Hsp18.8-CX*) were constitutively expressed in root, shoot and leaf. The constitutive expression of sHsps in vegetative organs implies that these proteins may be involved in house keeping activity of the cells. Overall, there is a considerable variation in the expression of sHsp genes under HT and development. Though *Hsp17.9B-CIX *and *Hsp16.6-CVIII *were induced by HT, the extent of induction was not as high as noted for CI sHsps. Expression of *Hsp18.8-CX *was not responsive to HT. In seed, moderate to negligible expression levels of *Hsp17.9B-CIX*, *Hsp16.6-CVIII *and *Hsp18.8-CX *were observed. The transcript of *Hsp17.8-CXI *was induced to a mild extent under HT. The expression of this gene was not noticed in any other developmental stage except in seed (albeit to low level). Multiplicity of these genes in cytoplasm may suggest functional redundancy of cytoplasmic sHsps. However, the expression profile results may also be considered as a support to hypothesize that these proteins perform diverse functions under stress and development.

All the organellar sHsps were induced by HT. *Hsp23.6-MII *showed constitutive expression in root, shoot and leaf. Differential expression of organellar sHsps was evident during various stages of anther development (Additional file 4) and seed. In *Arabidopsis*, peroxisomal and mitochondrial *Hsp26.5-MII *was expressed in seeds and none of the organellar sHsps were reported in any other development stage. Under stress conditions, sHsps reportedly function as chaperones protecting cellular proteins from denaturation and maintaining the protein homeostasis of the cells. Functional roles of sHsps under unstressed conditions have not been extensively worked out. Recently, it is proposed that these proteins may perform non-chaperone functions under constitutive conditions [8].

Acd proteins which seem to have diverged from sHsps during the course of evolution (Figure 1) showed expression patterns resembling CV and CVI sHsps. Most of the Acd genes were constitutively expressed in vegetative organs (Additional file 5). These genes were rather downregulated under HT. In this respect, Acd genes are similar in expression to CV sHsps of *Arabidopsis*. Cellular roles of Acd proteins are not very well established. OsAcd58 (OsSLT1) and its homologs in tobacco and *Arabidopsis *are shown to be constitutively expressed [45,46]. The C-terminal-truncated OsSLT1 exhibited ATP-independent chaperone activity and also provides enhanced thermotolerance to recombinant *E. coli *[45]. In contrast, AtSLT1 and NtSLT1 are implicated in Na^+ ^homeostasis [46]. Another Acd gene, AtAcd32.1 (which does not have homolog in rice) shows high level of constitutive expression and possesses chaperone activity *in vivo *[7]. Thus, it may appear that functionally Acds have role similar to constitutive sHsps or could be that these proteins are involved in specialized functions.

At the molecular level, it is proposed that the interplay of HSEs with corresponding trans-regulatory HSFs regulates the expression of Hsp genes. It is well documented that variations in the configuration of HSEs have a profound role in the expression of Hsp genes in stress and development [47]. The *in silico *analysis carried out in this study showed that promoters of almost all the sHsp genes harbor minimum one module of putative HSE. Various modules of HSEs have been shown to be functional in the promoters of sHsp genes in stress and embryogenesis [47,48]. Analysis of stress inducibility of three sHsp genes by promoter-GUS fusion revealed that promoter of *Hsp16.9A-CI *with imperfect HSE was induced 6.2 times and the promoters of *Hsp18.0-CI *and *Hsp17.4-CI*, both having perfect HSEs were induced 14.4 and 17.1 times, respectively [29]. However, expression of *Hsp16.9A-CI *was noted to be higher in comparison to *Hsp18.0-CI *and *Hsp17.4-CI *under similar stress conditions [29]. Experimental verification of sHsp promoters by deletion analysis may reflect the relevance of these putative HSEs in stress and development.

## Conclusion

This study makes contributions towards the genomic complexity and expression diversity of the sHsps gene family of rice. Through global transcript profiling by microarray and RT-PCR analysis, we have shown that most of the sHsps genes are highly upregulated in response to high temperature. We also noted that several sHsp genes are expressed constitutively in vegetative tissues and during panicle or seed development. Thus, we show that sHsps may be involved in cellular functions under non-stress and stress conditions as well as during developmental processes. It remains to be appreciated how the individual members of the sHsp gene family which have different pattern of expression depending on the organ, tissue and stage of development, are important in co-ordination of the overall heat shock response.

## Methods

### Screening of database and sequence analyses

Rice genome annotation database at TIGR was searched by keyword alpha crystallin protein, small heat shock protein and heat shock protein to predict genes containing conserved ACD region. Sequences obtained by keyword search were used as query in NCBI BLAST. Additionally the PS01031 domain containing entries of rice were searched at  (using taxonomic tree view of all Swiss-Prot/TrEMBL entries matching PS01031). This search returned 64 entries. Subsequently, the sequences were manually analyzed to identify and exclude duplicated annotations or sequences with conflict with genomic sequences in GenBank at the NCBI database. All predicted proteins were examined for Hsp20 domain in Prosite . The full length cDNA (FL-cDNA) of all predicted genes were searched at KOME database (knowledge-based Oryza molecular biological encyclopedia; ). Duplications of sHsp genes were investigated at TIGR by using segmental genome duplications page of rice . Specific targeting sequences were predicted with the PSORT program  and localization was predicted by Predotar  and TargetP or manual scrutiny for peroxisomal targeting. The prediction of transmembrane domains was performed with the TMHMM 2.0 program . Multiple sequence alignments were performed using the ClustalX1.83 . The phylogenetic tree was constructed using the neighbor-joining method and the bootstrap test carried out with 1000 iterations. One gene represented by Os05g23140 sequence was not included in this analysis as it is a retrotransposon. Molecular weight of the retrieved proteins was calculated using EditSeq of the DNASTAR program.

1.2 kb upstream region of sHsp genes was identified by BLAST in NCBI database and HSEs were searched in the PLACE . database. Further analysis to identify conserved motifs present in the promoter regions was performed using expectation maximization method MEME [49]. The program was set to output 7 motifs with minimum and maximum motif length of 10 and 25 bp, respectively. Distribution of motif occurrences was allowed to any number of repetitions in the given strand only. The MEME motifs were compared with the defined HSEs.

Two entries retrieved in BLAST analysis in NCBI have conflicting sequences with genomic sequence of *Hsp18.0 *(Os03g16030). These entries code for Hsp18.0 (accession- U83670, ORF- 483 bp, direct submission Guan and Lin, 1998) and Hsp17.8 (accession- X75616, ORF- 483 bp, direct submission Lin, 1996). Both these sequences mapped to chromosome 3 corresponding to locus Os03g16030 which codes for Hsp18 (161 aa) in rice genome. However, both sequences code for 160 aa sHsp because one amino acid at position 64 is missing compared to genomic sequence. Furthermore, both sequences have substitution of S to T at position 35 as compared to Hsp18 (161 aa). Similarly, for plastid Hsp there are 4 full length cDNA clones in database. AK120045, AK120048 and AK063618 have 723 bp ORF coding for of 240 aa whereas directly submitted clone AB020973 has an mRNA of 720 bp coding for 239 aa protein [30]. AK120048 and AK063618 perfectly match with the genomic sequence while AK120045 has one nucleotide mismatch and the AB020973 has 3 gaps and 3 mismatches with the genomic sequence.

Digital expression analysis of sHsps and Acd genes was performed using gene expression evidence search tool against the rice data available at TIGR  for different tissues in the following libraries: UV-C irradiated shoot (Acc#19038), mixed shoot (normalized library) (Acc#19041), supermix (Acc#19047), panicles mixture of one, two, three weeks after flowering (Acc#19050), root of seedlings (Acc#19053), flower (Acc#19057), callus (Acc#19058), after pollination embryo (Acc#19099), 40 days after pollination pistil (Acc#19121) and seed (Acc#19082).

### Growth conditions and stress treatment

Rice seeds [*O. sativa *L; cultivar Pusa basmati 1 (PB1) obtained from Indian Agricultural Research Institute, New Delhi, India] were treated with 70% alcohol for 5 min followed by rinsing 5–6 times with water. Seeds were soaked for two days in water and then grown on 1 cm layer of cotton in a tray. 7 d old seedlings were subjected to heat stress at various temperatures and time periods by placing them in beakers containing equal volume of water in water bath maintained at requisite temperatures. For cold stress, seedlings were placed for specified time in cold room maintained at 6 ± 2°C.

### RNA isolation and microarray

For analysis of transcript levels by RT-PCR, RNA was isolated from various tissues by TRI reagent (Sigma) as recommended by the manufacturer. RNA from dry seeds was isolated according to Singh et al [50]. 5 μg of total RNA was reverse transcribed with an oligo-dT primer using MMLV reverse transcriptase first-strand synthesis system for RT-PCR as recommended by the manufacturer (MBI, Fermentas) in a 50 μl reaction. For PCR, 1 μl cDNA was taken in a reaction volume of 25 μl using gene specific primers for 25 cycles (List of primers in Additional file 2).

For microarray analysis, RNA was isolated from rice leaves (control at 26°C, stressed at 42°C for 10 min, 1 h and 30 min recovery after 1 h stress) by RNeasy plant mini kit (Qiagen). The integrity of RNA was checked by agarose gel electrophoresis and quality was checked spectrophotometrically. Expression profiling was conducted with the 60K Rice Whole Genome Microarray (information available at ; GreenGene Biotech). In total, 60,727 oligomers were designed from gene-specific regions of both japonica and indica subspecies. These include 58,417 from known and predicted genes and 66 randomized DNA oligomers. Oligomer sequences were extracted by Qiagen-Operon based on rice genome information from the Beijing Genomics Institute. Oligomers were synthesized and purified by Qiagen-Operon and spotted on SuperAmine slides using the facilities of Dr. David Galbraith at the University of Arizona . A set of two slides of the 60K microarray has 64,896 spot addresses. Each slide is formatted with 48 (12 × 4) blocks composed of 676 (26 × 26) spots. Blank spots (4099) were also included for easy scanning of the alignment. Each oligomer 70 nucleotides long with an average Tm of 78°C was printed in each spot address with a diameter of 100 μm.

Non-correlation of signal and background intensities was confirmed by plotting base 2 log background intensity in x-axis and base 2 log intensity subtracted from background intensity on the y-axis. Before normalization, the normal distribution and linear relations of Cy3 and Cy5 intensities were tested by qqplot and a linear regression model, respectively, in R statistical language. The spatial effects on the chip during the hybridization process were checked with spatial func in the sma package. The variance differences between Cy3 and Cy5 intensities within the microarray were tested with the Student's t-test under the assumption of both uniform and non-uniform variances. One- and two-way analyses of variance of the signal intensity differences between microarrays were performed. Median pixel intensities were transformed as log ratios with base 2 and then adjusted by block-by-block Lowess normalization for each slide [51]. To improve the specificity of statistical hypothesis in low-intensity regions, following empirical criteria was adopted: a spot was selected if it was not flagged for its morphology, the diameter was larger than 51 pixels, and the intensities of both signals were higher than 500. Data presented are based on three biological replicates at indicated times.

## Abbreviations

sHsp: small heat shock protein; ACD: alpha crystallin domain; HT: high temperature; HSE: heat shock element; HSF: heat shock factor; PCR: polymerase chain reaction.

## Authors' contributions

NS and AG planned and designed the study. NS performed computational analysis, executed the experiments, generated the figures and drafted the manuscript. NS, YKK and AG designed the microarray experiment. YKK participated in the microarray and its data analysis. AG contributed to the discussions and preparation of the manuscript. All authors read and approved the final manuscript.

## Supplementary Material

Additional file 1 **(A) Alignment of amino acid sequences of ACDs of sHsps and Acds used for generating phylogenetic tree in Figure **1. (B) Alignment of amino acid sequences of sHsp genes of rice and other plant species used for constructing phylogenetic tree in Figure 2. Click here for file

Additional file 2 **Supplemental Table 1.** Predicted cellular location of organellar sHsps of rice. Supplemental Table 2. List of primer sets for RT- PCR. Click here for file

Additional file 3 **Amino acid sequence alignment of new subfamilies.** Click here for file

Additional file 4 **Supplemental Figure 2.** Expression pattern of sHsps during anther development in rice. Click here for file

Additional file 5 **Supplemental figure 3(A).** Expression pattern of Acd genes during development stages of rice plant. Supplemental figure 3(B). Microarray based expression profiles of Acd genes of rice under heat stress. Click here for file

Additional file 6 **Supplemental Table 3.** MEME analysis of promoters of sHsp genes. Click here for file

Additional file 7 **Manual analysis of promoters of sHsp genes.** Click here for file
