# Supplementary material for: Rice sHsp genes: genomic organization and expression profiling under stress and development
Source: BMC Genomics. 2009 Aug 24;10:393. doi: 10.1186/1471-2164-10-393 (PMC2746236; doi:10.1186/1471-2164-10-393)
Supplement: Additional file 1 — (A) Alignment of amino acid sequences of ACDs of sHsps and Acds used for generating phylogenetic tree in Figure 1. (B) Alignment of amino acid sequences of sHsp genes of rice and other plant species used for constructing phylogenetic tree in Figure 2. [file 1471-2164-10-393-S1.rtf]

Additional file 1
(A)   Alignment of amino acid sequences of ACDs of sHsps and Acds used for generating phylogenetic tree in Figure 1.


               ....|....| ....|....| ....|....| ....|....| ....|....| ....|....| 
                   5          15         25         35         45         55                     
Hsp18.0-CI     ---------- -DWKET--PE AHVFKADVP- -GLKKEEVKV EVEDG---NV LQISGER--- 
Hsp17.4-CI     ---------- -DWKET--PE AHVFKADVP- -GLKKEEVKV EVEDG---NV LQISGER--- 
Hsp17.7-CI     ---------- -DWKET--PE VHVFKADVP- -GLKKEEVKV EVDDG---NI LQISGER--- 
Hsp17.9A-CI    ---------- -DWKET--PE AHVFKADVP- -GLKKEEVKV EVDDG---NI LQISGER--- 
Hsp16.9B-CI    ---------- -DWKET--PE SHVFKADLP- -GVKKEEVKV EVEEG---NV LVISGQR--- 
Hsp16.9A-CI    ---------- -DWKET--PE SHVFKADLP- -GVKKEEVKV EVEEG---NV LVISGQR--- 
Hsp16.9C-CI    ---------- -DWKET--PE SHVFKADLP- -GVKKEEVKV EVEEG---NV LVISGQR--- 
AtHsp17.6A-CI  ---------- -DWKET--AE AHVFKADLP- -GMKKEEVKV EIEDD---SV LKISGER--- 
Hsp16.6-CVIII  ---------- -DWKET--PT AHVFTADLP- -GVRKDQAKV EVEDG---GV LVISGER--- 
Hsp18.8-CX     ---------- -DWKET--AA AHVFMADMP- -GVRREEVRV EVEEE---KV LRISGQR--- 
Hsp17.9B-CIX   ---------- -ESRET--AE AYVFRADLPA -GVKKEEVRV EVDEG---NV LVITGER--- 
Hsp23.2-ER     ---------- -DWRET--GD AHEVVVDVP- -GMRKEDLRV EVEDN---RV LRISGER--- 
AtHsp22-ER     ---------- -DWKET--AE GHEIMLDIP- -GLKKDEVKI EVEEN---GV LRVSGER--- 
Hsp21.8-ER     ---------- -DWKET--PE AHVVTVDVP- -GVRRGDVRV EVDEAS--RV LRVSGER--- 
Hsp17.8-CXI    ---------- -DWRET--PV AHVFEMDLP- -GLAKDQVAV EVVDG---HI LRVRAGG--- 
Hsp16.0-Px     ---------- -DWVET--PT SHVLRINVP- -GLGKDDVKV QVEDG---NV LTVRGAAPHA 
At15.7Px       ---------- -DWMES--NN SHIFKINVP- -GYNKEDIKV QIEEG---NV LSIRGEG--- 
Hsp18.8-CV     ---------- ---ISL--ID TKRGMVGL-- -GVRKEEIRV EVEDA---MY LVIRT----- 
AtHsp15.4-CI(r)---------- -RWSQS--PD SHTFSVDLP- -GLRKEEIKV EIEDS---IY LIIRT----- 
AtHsp17.6-CII  ---------- -DVIEH--PN AYAFVVDMP- -GIKGDEIKV QVEND---NV LVVSG----- 
AtHsp17.7-CII  ---------- -DVIEH--PD AYVFAVDMP- -GIKGDEIQV QIENE---NV LVVSG----- 
Hsp18.0-CII    ---------- -DVKDL--PG AYAFVVDMP- -GLKSSDIKV QVEEE---RL LVISG----- 
Hsp19.0-CII    ---------- -DVKELRASG ALVLAVDMP- -GVAPADVRV EVEDG---NV LAISG----- 
Hsp18.6-CIII   ---------- -DIMET--PG EYAFVLDVP- -GLSKSDIQV TLEED---RV LVMKSSNGAG 
AtHsp17.4-CIII ---------- -DILES--PK EYIFYLDIP- -GISKSDIQV TVEEE---RT LVIKS----- 
Acd58.3        ---------- -SIYE--DDQ GYLIMVSLP- -FVDQQKVKV SWRNSLTHGI VKILCVSTAQ 
AtAcd55.8      ---------- -TVYE--DEE AYLVVITLP- -FVDLNTVKV SWRNNITNGI VKVTGLSTSR 
AtAcd39.4      ---------- -DIGE--CDD AYLFRVSLP- -GVKRDERYF SCEVE-DNGK VLVRGVTTTG 
AtAcd22.3      ---------- -DIAE--SED SYYFRVALP- -GVSRDEKEF SCEIE-PDGK IMIKGATTTG 
AtAcd15.5      ---------- -DIGV--SEV AYIFRVSLP- -GIEKNQDKI KCEIQ-REGR VCIQGVIP-E 
AtAcd28.7      ---------- -LSRE--TNS VFFLILHLK- -GFKKDGIDI EINKE--GNL IKISGRKQVE 
AtAcd216.9     ---------- ---------- ---------- ---------- ---------- ---------- 
Hsp22.3-CVI    ---------- -DWAE--TDS EYYLRADIP- -GGRKCDVEV SGDDA--MRV VDVSGLWRAA 
AtHsp21.7CI(r) ---------- -DWSQ--TDQ AYVLKSDIP- -VVGKNNVQV YVDIN--GRV MEISGQWNSN 
Acd52.9        ---------- -NVRQ--TKD CFEIYALVP- -GLLREEVHV QSDPA---GR LVITGDPE-- 
Acd50.4        ------DWVK INVRR--TKD CYEVYALVP- -GLLREEVHV QSDPA---GR LIVTGEPE-- 
AtAcd86.6      ---------- -NVQR--TQD CFEVYALVP- -GLVREEVRV QSDPA---GR LVISGEPE-- 
AtAcd48        ---------- -NVRE--TKD CFEIFALVP- -GLLREEVRV QSDPA---GR LVIAGQPE-- 
AtAcd44.3      ---------- -NVKE--SKD SFEIFALVP- -GLLRKEVRI QSDPA---GK VVITGQPE-- 
Acd29.4        ---------- -EWLD--AGA HYLLRVNVP- -EFKKEELQV HVDP---AGR LTVRGQH--G 
Acd34.9        ---------- -EWLD--NAN NFLLRLYLT- -GFKKEDFRV QVDG---TGK LTVRGQRPAA 
Acd18.1        ------DLRE QVWLDG-AAA GYVVRLDIA- -GFSKDEVDV RVNGA--TGR VTVLGQRPAA 
Acd18.0        ---------- -KMVR--EPP THTLTIDLSA KGYKKEHIKV QLVRS--RRR LVVSGECPVA 
Acd22.6        ---------- ---VE--EAG KKVLQINLSA AGFKKEQLRV QIDN---HGK LRISGERQVS 
AtAcd16.6      ---------- ---------- ---------- -GLKKEHLKI QINN---SGV LTITGGCPVD 
Acd19.4        ---------- -EWIH--GDE FDTLVLDVT- -GFSKDHLKV QVEA---SGS LRISGERAVN 
Acd41.4        ---------- -EWTR--TED ADTLVVDVS- -GFRKEELKV LYNT---SRK LKVAGERRAD 
Acd31.8        ---------- -EWSR--SAE ADAVKISLP- -GFKREEIRV -LVDN--HGH LRTRGERPVA 
AtAcd41.3      ---------- -EWKD--QPE ATILNIDLT- -GFAKEQMKV TYVHS--SKM IRVTGERPLA 
AtAcd28.1      -----EFEPA TRWTS--EPD AEVLVADLP- -GFKKEQLKV -SVTA--TRK LRLTGERPTG 
AtAcd25.1      ---------- ---------- ---LTIYLP- -GFRKEQLKV -QVTT--TRK LRVMGDRPAG 
Hsp26.7-P      ---------- -DVMED--DK EVRMRFDMP- -GLSREEVKV MVED----DA LVIRGEHK-- 
Acd19.1        ---------- -DIMDD--DK RGEIRFDML- -GLSREDVKV MVED----NM LVIRGEHS-- 
AtHsp25.3-P    ---------- -DIKEE--EH EIKMRFDMP- -GLSKEDVKI SVED----NV LVIKGEQK-- 
Hsp26.2-MI     ---------- -VAKED--DD AVHLKVSMP- -GLGKEHVKV WAEQ----NS LVIKGEGE-- 
Hsp24.0-MI     ---------- -NAKES--EE ALHLRVDMP- -GLGKEHVKV WAEQ----NS LVIKGEGE-- 
AtHSP23.6M     ---------- -DIKEK--DD ALYLRIDMP- -GLSREDVKL ALEQ----DT LVIRGEGK-- 
AtHsp23.5-M    ---------- -NVKEK--DD ALHLRIDMP- -GLSREDVKL ALEQ----NT LVIRGEGE-- 
Hsp23.6-MII    ---------- -KVRED--EE RYRLRFEVP- -GLGKDDVRV YVDD----GV LAIHGEKRDV 
AtHsp26.5-P(r) ---------- -QVKEQ--DD CYKLRYEVP- -GLTKEDVKI TVND----GI LTIKGDHK-- 
AtAcd27.7      ---------- ESKQLP--NG KLFVRADMP- -GVPKENFTV SVTN----GR VKVTGQAP-- 
AtAcd25.4      ---------- -SVKLP--NG KLFVRADMP- -GVPKENFTV SVTN----GR VKVTGEAP-- 
AtAcd81.4      ------EMAY ESKQLQ--NG GLYVRVDMP- -GVPKENFTV AVMN----GR VRVTGEAP-- 
AtAcd57.7      SVLEGPMMPY ETKQLS--NG GLYMRVDMP- -GVPSEKFMV AVDGD---GV VTIMGRAP-- 
Acd50.7        ---------- -ATSDT--DA AFLVLAHLP- -GYDKEEVEV VVGDG-GREV GVVVGARKDD 
Acd21.0        ---------- -QVAEN--KD EVSLWFDVP- -GLSPADLIV EIDED----V LVIKKKKKAS 
Acd38.4        ---------- ---------- -VQLWLQVP- -GLTEDDLEI TTTD----EL LEIKRKAG-- 
Acd21.5        ---------- --ITED--DD YVKLWFHVG- -EIDREKLKV RIEHD--TVL LVSYGGAG-- 
AtHsp14.7-P(r) ---------- -EVTET--KK SCVTRVDMP- -GCPESDLTY WVDAN---NV HFFADEP--- 
AtAcd32.1-Px   ---------- -NVAES--TH SYVVAIELP- -GASINDIRV EVDN----TN LTVTGRRTSI 
AtAcd54.2      ---------- -QWTNS--GS SIFLYVNLP- -GFYRDQIEI KKDER--TRT VQIQGQRP-- 
Acd30.2        ---------- -DIPTG--GK HVQMKFHVP- ETIDPAKLHV SIKDR----D LIVKAEDK-- 

               ....|....| ....|....| ....|....| ....|....| ....|....| ....|....| 
                   65         75         85         95        105        115                   
Hsp18.0-CI     --SKEQ---E EKTD-----K WHRVER---- SSG--KFLRR FRLP--ENTK PEQ-IKASME 
Hsp17.4-CI     --IKEQ---E EKTD-----K WHRVER---- SSG--KFLRR FRLP--ENTK PEQ-IKASME 
Hsp17.7-CI     --SREQ---E EKSD-----K WHRVER---- SSG--KFLRR FRLP--ENTK PEQ-IKASME 
Hsp17.9A-CI    --NKEQ---E EKTD-----Q WHRVER---- SSG--KFLRR FRLP--DNAK PEQ-IKASME 
Hsp16.9B-CI    --SKEK---E DKND-----K WHRVER---- SSG--QFMRR FRLP--ENAK VDQ-VKAGME 
Hsp16.9A-CI    --SKEK---E DKND-----K WHRVER---- SSG--QFMRR FRLP--ENAK VDQ-VKAGLE 
Hsp16.9C-CI    --SKEK---E DKND-----K WHRVER---- SSG--QFMRR FRLP--ENAK VDQ-VKASME 
AtHsp17.6A-CI  --HVEK---E EKQD-----T WHRVER---- SSG--GFSRK FRLP--ENVK MDQ-VKASME 
Hsp16.6-CVIII  --AREEDV-D GKNDE----R WHHVER---- SSG--KFQRR FRLP--RGAR VDQ-VSASMD 
Hsp18.8-CX     --ARAA---E EKGE-----R WHRVER---- SSE--RFVRT VRLP--PNAN TDG-VHAALD 
Hsp17.9B-CIX   --SVRR---E EKGQ-----R SHHIER---- SCA--TFFGR FHLP--DDAV VDL-VRASMD 
Hsp23.2-ER     --RREETT-E QKGG---GDH WHREER---- SYG--RFWRQ LRLP--DNAD LDS-IAASLD 
AtHsp22-ER     --KREE---E KK-----GDQ WHRVER---- SYG--KFWRQ FKLP--DNVD MES-VKAKLE 
Hsp21.8-ER     --RRAGAA-E EEEGERDGVR WHRAER---- AAG--RFWRR FRMP--PGAD VGR-VAARLD 
Hsp17.8-CXI    --EHEDANNA AKAG---KAS GEEEEE---- NDGV-RWHCR ERAAG-RRRA ADE-ASARMA 
Hsp16.0-Px     AAEKER---E REKD----VV WHVAER---- -GRP-EFARE VALP--AEVR VEQ-IRASVD 
At15.7Px       --IKE----E KKEN----LV WHVAEREAFS GGGS-EFLRR IELP--ENVK VDQ-VKAYVE 
Hsp18.8-CV     ---------E LDDG-----G DGDGGG---- GGGRRSFARK FRLP--AMVD ADG-ISAEYT 
AtHsp15.4-CI(r)---------E ATPM-----S PPDQP----- ---LKTFKRK FRLP--ESID MIG-ISAGYE 
AtHsp17.6-CII  --ERQR---E NKEN--EGVK YVRMER---- RMG--KFMRK FQLP--ENAD LDK-ISAVCH 
AtHsp17.7-CII  --KRQR---D NKEN--EGVK FVRMER---- RMG--KFMRK FQLP--DNAD LEK-ISAACN 
Hsp18.0-CII    --ERRRGGGE EEKE--ESCK YLRMER---- RMG--KFMRK FVLP--DNAD VDK-ISAVCQ 
Hsp19.0-CII    --ERRRPAGD GDDGG-EGVK YLRMER---- RMG--KFMRR FPLP--ESAD LDG-VRAEYK 
Hsp18.6-CIII   NGKRKR---E EEEGE---CK YIRLER---- RASPRAFARK FRLP--EDAD TGG-ISARCE 
AtHsp17.4-CIII NGKRKR---D DDESE-EGSK YIRLER---- RLA-QNLVKK FRLP--EDAD MAS-VTAKYQ 
Acd58.3        TPYIRRHDRV FKLAD-PMPE HCPHGE---- ------FVRE IPLAT--RIP EDAKLEAYFD 
AtAcd55.8      ASFVKRRDRT FKLVD-QMAE HCPPGE---- ------FMRE IQLPN--RIP EEANIEAYFD 
AtAcd39.4      GKRVKRYSHV FEMQ---TRS LCPPGN---- ------FSVS FRLPG--PVH PHE-FSGNFG 
AtAcd22.3      EQTVCKHNQI FKML---TQN LCPPGH---- ------FTIN FQLPG--PVS NEE-FNGNFG 
AtAcd15.5      IAIPSDTGCL YRMQ---VQQ LCPPGP---- ------FSIT FNLPG--QVD PRL-FSPNFR 
AtAcd28.7      EMVLVK---- WVEW----KK ETEIKE---- ------FKKV FRIPD--IVI LDK-IKARFN 
AtAcd216.9     ---------- --------RK EVEFSV---- ------FKKV FRIPD--TVD LDK-IKARFD 
Hsp22.3-CVI    PPPPPPDGRD WRAG-----R WWEHG----- ------FVRR VELPE--DAD WRK-VEAFFD 
AtHsp21.7CI(r) KK--AATNSD WRSG-----R WWEHG----- ------YVRR LELPS--DAD AKY-SEAFLS 
Acd52.9        ---------- -QPD-----N PWGITP---- ------FKKV VNLPL--RID PHQ-TSAVVT 
Acd50.4        ---------- -QLD-----N PWGVTP---- ------FKKV ISLPS--RID PHQ-TSAVVT 
AtAcd86.6      ---------- -NPM-----N PWGATP---- ------FKKV VSLPT--RID PHH-TSAVVT 
AtAcd48        ---------- -QLD-----N PWGITP---- ------FKKV VNFPA--RID PLH-TSAVVS 
AtAcd44.3      ---------- -QLD-----N PWGITP---- ------FKKI VDLSA--RID PLH-TSAVMS 
Acd29.4        G--------- ---------- ----LR---- ------LNKV FQLPP--TCN LDA-ITGRLE 
Acd34.9        GS-------- ---------- -KHNTR---- ------FHKV FQLPS--NAN IDD-ITGRFE 
Acd18.1        AG-------- ---------- --PHVR---- ------LRRV IQLPP--TAD SDR-VAARFV 
Acd18.0        GET------- ---------- -NRWSR---- ------FRLQ FPVPD--GCD LKA-IQARLH 
Acd22.6        G--------- ---------- -NRWSR---- ------FHKD FQVPD--DCN AGD-VRARFD 
AtAcd16.6      Q--------- ---------- -TKTIR---- ------FMKE TKVAK--DCK RNE-IRAKFS 
Acd19.4        GGG------- ---------- -RHWLH---- ------FLKR FDLPPGAADD ASA-IKVQLD 
Acd41.4        GG-------- ---------- --QWAR---- ------FLKM FPVPR--SCD AGA-IRAVMD 
Acd31.8        G--------- ---------- -NRWSR---- ------FQKD FQLPA--DCN VDG-IRAKFE 
AtAcd41.3      N--------- ---------- -RKWSR---- ------FNEV FTVPQ--NCL VDK-IHGSFK 
AtAcd28.1      G--------- ---------- -NKWIR---- ------FHQE IPVPL--TVD IDS-VSAMFK 
AtAcd25.1      A--------- ---------- -NKWIR---- ------FRKE FPIPP--NID VDS-VSAKFE 
Hsp26.7-P      KEEGE-GAEG SGDGW----W KERSVSS--- ------YDMR LALP--DECD K-SKVRAELK 
Acd19.1        KEEKERGAPT MGGG------ --RSAA---- ---------- ---------- ---------- 
AtHsp25.3-P    KEDSD----D SWSG------ --RSVSS--- ------YGTR LQLP--DNCE K-DKIKAELK 
Hsp26.2-MI     KDP---EDDA DAAP------ -PRYTR---- ---------R IELP--ADAF KMDKIKAEMK 
Hsp24.0-MI     KEA---GEDE GAAP------ -ARYSG---- ---------R IELA--PEVY RMDQIKAEMK 
AtHSP23.6M     NEEDG-GEEG ESGN------ -RRFTS---- ---------R IGLP--DKIY KIDEIKAEMK 
AtHsp23.5-M    TEE---GEDV SGDG------ -RRFTS---- ---------R IELP--EKVY KTDEIKAEMK 
Hsp23.6-MII    VEEDR-GRDG DGECW----A -AATY----- -------HAG LLLP--EDAV A-EGITAEVR 
AtHsp26.5-P(r) AEEEK-GSPE EDEYW----S -SKSYGY--- ------YNTS LSLP--DDAK V-EDIKAELK 
AtAcd27.7      ------AVSH DSSG------ --RFYSG--- ------DVAM LSTP--VDIP S-RRIKTIAK 
AtAcd25.4      ------ALSH DSSG------ --RFYSG--- ------DGAM LSTP--VDIP S-RRIKTIAK 
AtAcd81.4      ------AISH DSSG------ --RFYTG--- ------DVAM LSTP--FDIP I-RKIKIIAK 
AtAcd57.7      ------VTMH DTSG------ --RTYVA--- ------KVAN VPRG--YDG- --GRIKLVPK 
Acd50.7        AFAVEAAVVG RRLR----VA HRQVVEG--- ------FCRV FDVPP--GVE VGR-ITVGFE 
Acd21.0        PKSNYNTPTS GAIADHQEAT ADEFSGG--- -----GIYAR LLLP--AGYS REG-VQAKLT 
Acd38.4        ---------R GDPR-----R LDDVQGVG-- -----SFHLR LLLT---KEF VSSQVTAELK 
Acd21.5        ---------- DETS------ -TPANS---- ------LDVR LLLPN-KPYD TAK-VEAELT 
AtHsp14.7-P(r) ---------- -AMP-----E YENAGR---- -----KYGGS MIFNP-EAYD VKK-TKVKLI 
AtAcd32.1-Px   CQKVDAGTKA SILG----YH KQEILQG--- -----PFKVS WPLPS--NVN KDN-VSAEFM 
AtAcd54.2      ---------- --LS------ AQTKAR---- ------FSEA YRVP--DTCD MTK-LSTSFS 
Acd30.2        ---------V EKPD------ --GVSR---- ----FYYYKR TTLP--ENTD FKS-LKCNYD 

                ....|....| ....|....| ....|....| ....|
                   125        135        145        155     
Hsp18.0-CI      NG----VLTV TVPKEE-PKK PDVKSIQVT- -----
Hsp17.4-CI      NG----VLTV TVPKEE-PKK PDVKSIQIT- -----
Hsp17.7-CI      NG----VLTV TVPKEE-PKK PDVKSIQIS- -----
Hsp17.9A-CI     NG----VLTV TVPKEE-AKK PDVKSIQIS- -----
Hsp16.9B-CI     NG----VLTV TVPKAE-VKK PEVKAIEIS- -----
Hsp16.9A-CI     NG----VLTV TVPKAE-VKK PEVKAIEIS- -----
Hsp16.9C-CI     NG----VLTV TVPKAE-VKK PEVKAIEIS- -----
AtHsp17.6A-CI   NG----VLTV TVPK------ ---------- -----
Hsp16.6-CVIII   NG----VLTV TVPKEE-TKK PQLKAIPIS- -----
Hsp18.8-CX      NG----VLTI TIPKDN-DRK PHARIIPIT- -----
Hsp17.9B-CIX    GG----MLTV TVPKVV-TDK Q--PAIAAA- -----
Hsp23.2-ER      NG----VLTV RFRKLAPDQI KGPRVVGIA- -----
AtHsp22-ER      NG----VLTI NLTK------ ---------- -----
Hsp21.8-ER      DG----VLTV TVPKVPGHRG REPRVVAID- -----
Hsp17.8-CXI     DG----VLTV TVPKRKGKKR HAGNGKAAGD DKPVC
Hsp16.0-Px      NG----VLTV VVPKEPAPAR PRTRPIAVS- -----
At15.7Px        NG----VLTV VVPK------ ---------- -----
Hsp18.8-CV      HG----VLRV TVPRLHTRAR PVV------- -----
AtHsp15.4-CI(r) DG----VLTV IVPK------ ---------- -----
AtHsp17.6-CII   DG----VLKV TVQ------- ---------- -----
AtHsp17.7-CII   DG----VLKV TIPK------ ---------- -----
Hsp18.0-CII     DG----VLTV TVEKLPPPE- ---------- -----
Hsp19.0-CII     DG----VLTV TVDKKPPPEP KKPRVVEVK- -----
Hsp18.6-CIII    NG----VLTV TVKKRPPPEK KTKSVQVT-- -----
AtHsp17.4-CIII  EG----VLTV VIKK------ ---------- -----
Acd58.3         EAAA--VLEI MVPKRGNEPE EHEVRV---- -----
AtAcd55.8       GTGP--VLEI VVPK------ ---------- -----
AtAcd39.4       TDG---ILEG VVMK------ ---------- -----
AtAcd22.3       SDG---VLEG VVKK------ ---------- -----
AtAcd15.5       SDG---IFEV VVVK------ ---------- -----
AtAcd28.7       EEDG--TLTV TMPK------ ---------- -----
AtAcd216.9      DDDA--TLTI TMPK------ ---------- -----
Hsp22.3-CVI     DGE--GLLEI KVPK------ ---------- -----
AtHsp21.7CI(r) NNDDYSFLEI RIPK------ ---------- -----
Acd52.9        LHG---QLFV RAPF------ ---------- -----


Additional file 1 
(B)  Alignment of amino acid sequence of sHsp genes of rice and other plant species used for constructing phylogenetic tree in Figure 2.    

             ....|....| ....|....| ....|....| ....|....| ....|....| ....|....| 
                 5          15         25         35         45         55                     
Le26.2       MAYTSLTSSP LVSNVSVGGT SKINNNKVSA PCSVFVPSMR ---RPTTRLV -ARAT----- 
Vv25.0       --MASLKAFP FSSSPLATHK PSLSKGVAGA PCSAFFPSSR HGGRSRLALV RAEAT----- 
At25.3       --MASTLSFA ASALCSPLAP SPSVSSKSAT PFSVSFP--- ---RKIPSRI RAQDQ----- 
Hsp26.7-P    ----MAAPFA LVSRVSPAAR LPIRAAWRRA RPTVGLPSSG ---RARQLAV ASAAQ----- 
Ta26.7       -MADANAPFA LVSRLSPAAR LPIR-AWRAA RPAP-LSTGG ---RTRPLSV ASAAQ----- 
Zm26.4       ---MAAAPFA IAGRLSPVAR LPVR-AWR-- -PAHGFASSG ---RARSLAV ASAAQ----- 
At23.6-M     --MAS--ALA LK-------R LLSSS----- ----IAPRSR --SVLRP-AV SSRL------ 
At23.5-M     --MASSSALA LR-------R LLSSSTV--- ----AVPRAL --RAVRPVAA SSRL------ 
Hsp26.2-MI   --MASTVALK GRPLATLLRQ LLAADAPPAA TGRPVAAAPA --ASGKPVTA PAAATATNAA 
Hsp24.0-MI   --MASIVASK RIPLFRLVEQ LLAASP---- -----AQGAA --SALRPVAV AGGS------ 
Zm22.8       ---MALARLC LNRAIAGRAQ ALARPALAAA PATDPKLHS- -------LLS TSAADSAAS- 
Hsp23.6-MII  ---MALARQC LSKRLAA-GC ALARPLHAAS PVAAAAANSH GPLNFRALFS SAGADAAATT 
At26.5       ---MALARLA LRNLQQKL-- --SPSLMGQS -CERGLVGNR HNPMKLNRFM AT--SAGEQ- 
Vv26.3       ---MALARLA LRSLQQRVPS SSSSSLLSPS LSERALTGQR WGPEIVKRFS ATPDAASDK- 
So17.4       ---------- ---------- ---------- ---------- ----MSLV-- ---------- 
Sb17.4       ---------- ---------- ---------- ---------- ----MSLV-- ---------- 
Zm17.4       ---------- ---------- ---------- ---------- ----MSLV-- ---------- 
Cd16.6       ---------- ---------- ---------- ---------- ----MSLV-- ---------- 
Hsp17.9B-CIX ---------- ---------- ---------- ---------- ----MSLV-- ---------- 
Zm16.6       ---------- ---------- ---------- ---------- ----MSPA-- ---------- 
So16.5       ---------- ---------- ---------- ---------- ----MSLV-- ---------- 
Hsp16.6-CVIII---------- ---------- ---------- ---------- ----MSLV-- ---------- 
Hsp16.9B-CI  ---------- ---------- ---------- ---------- ----MSLV-- ---------- 
Hsp16.9A-CI  ---------- ---------- ---------- ---------- ----MSLV-- ---------- 
Hsp16.9C-CI  ---------- ---------- ---------- ---------- ----MSLV-- ---------- 
Zm17.2       ---------- ---------- ---------- ---------- ----MSLV-- ---------- 
Ta16.9B      ---------- ---------- ---------- ---------- ----MSIV-- ---------- 
Hsp17.9A-CI  ---------- ---------- ---------- ---------- ----MSLI-- ---------- 
Hsp18.0-CI   ---------- ---------- ---------- ---------- ----MSLI-- ---------- 
Hsp17.4-CI   ---------- ---------- ---------- ---------- ----MSMI-- ---------- 
Hsp17.7-CI   ---------- ---------- ---------- ---------- ----MSLI-- ---------- 
Zm17.9       ---------- ---------- ---------- ---------- ----MSLI-- ---------- 
At17.4CI     ---------- ---------- ---------- ---------- ----MSLVP- ---------- 
Ta20.1       ---------- ---------- ---------- ---------- ----MSMISS MLGRKQPPPQ 
Hv19.3       ---------- ---------- ---------- ---------- ----MSMISN MLGRRQPPP- 
Ps19.8       ---------- ---------- ---------- ---------- ----MSMISS MLGRRQPPAQ 
Hsp18.8-CX   ---------- ---------- ---------- ---------- ----MSMITS MLGRK----- 
Vv16.4       ---------- ---------- ---------- ---------- ----MSMLS- ---------- 
Pt17.6       ---------- ---------- ---------- ---------- ----MSLLHS ---------- 
Hsp17.8-CXI  ---------- ---------- ---------- ---------- ----MSLVLS ---------- 
Zm22.7       ---------- ---------- ---------- MADAVLFSRK ASASAAVVVL AVAVVTMACL 
Hsp21.8-ER   ---------- ---------- ---------- MA-------- AVAEREVLGM VAAVAAMVVM 
Vv21.3       ---------- ---------- ---------- ---------- -MAKPSIIPI SLFLAAMAAV 
At22         ---------- ---------- ---------- ---------- ---MMKHLLS IFFIGALLLG 
Hsp23.2-ER   ---------- ---------- ---------- ---------- -MASMRTAAA AAMLACIAVV 
Hv17.1       ---------- ---------- --------MD ---------- ---------- ---------- 
Hsp18.8-CV   ---------- ---------- --------MD YY-------- ---------- ---------- 
Zm17.2       ---------- ---------- --------MD YS-------- ---------- ---------- 
Vv15.7       ---------- ---------- --------MD ---------- ---------- ---------- 
At15.4       ---------- ---------- --------MD ---------- ---------- ---------- 
Lp16.1CIII   ---------- ---------- --------MS T--------- ---------- ---------- 
At17.4CIII   ---------- ---------- --------MS ---------- ---------- ---------- 
Hsp18.6-CIII ---------- ---------- --------MT EL-------- ---------- ---------- 
At17.6-CII   ---------- ---------- --------MD LG-------- ---------- ---------- 
At17.7       ---------- ---------- ------MDLE FG-------- ---------- ---------- 
Hsp18.0-CII  ---------- ---------- --------ME SA-------- ---------- ---------- 
Hsp19.0-CII  ---------- ---------- --------ME LS-------- ---------- ---------- 
At15.7       ---------- ---------- ---------- ---------- ---------- ---------- 
Gm16.2       ---------- ---------- ---------- ---------- ---------- ---------- 
Hsp16.0-Px   ---------- ---------- ---------- ---------- ---------- ---------- 
Vv22.4       ---------- MTSSK---QL EVQSEDR--- TPQKWCVSLR EDKFEAFLSQ GN-------- 
At21.7       ---------- MTSSSGSLKL EIHTDDK--- TPGKWSVPLG DDVFRRFLSG GG-------- 
Hsp22.3-CVI  ---------- MPPRRG-IEV RQAVGDG--- AAPRWRMSLL ENTFSSFLQS IGGGVAADG- 
Zm21.9       ---------- MPGRRA-IDV RLLPGGGDAA AAPKWRMSLL ENTFSGFLQG AG----AD-- 
Clustal Co                                                                     

             ....|....| ....|....| ....|....| ....|....| ....|....| ....|....| 
                 65         75         85         95        105        115                   
Le26.2       -----GDNKD TSVDVHHSSA QGGNN--QGT AVERRPT-RM ALD------- -VSPFGVLDP 
Vv25.0       -----GENKD ASLDVQ---V HQGNK--GAT AVERQPR-RL ALD------- -ISPFGLLDP 
At25.3       -----REN-- -SIDVVQQGQ QKGN---QGS SVEKRPQQRL TMD------- -VSPFGLLDP 
Hsp26.7-P    -----ENRDN TAVDVHVN-- QDGGN-QQGN AVQRRPRRSS ALDG------ -ISPFGLVDP 
Ta26.7       -----ENRDN -SVDVQVSQA QNAGN-QQGN AVQRRPRR-A GFD------- -ISPFGLVDP 
Zm26.4       -----ENRDN -SVDVQVS-- QNGGNRQQGN AVQRRPRRAT ALDI------ SPSPFGLVDP 
At23.6-M     -----FNTN- -AVRSYDD-- --DGENGDGV DLYRRSVPRR RGDF------ FS--DVF-DP 
At23.5-M     -----FNTN- -AARNYED-- --GVDRN--- HHSNRHVSRH GGDF------ FS--HIL-DP 
Hsp26.2-MI   SR-RLYNTEG APLRRYDVVD ESGTDSGDEY DATDDGRRLT VPFF------ FSASDVL-DP 
Hsp24.0-MI   ---RAYNTG- AQLRRHER-D ESDDDSGRGY DTRRPTRDAT MPAF------ FS--DVFRDP 
Zm22.8       -------GEA NRRDVAVSER SAPNRRWAWR DLRD------ ---------- FTPFS---LV 
Hsp23.6-MII  GGCAPAKGDG HSREVAVVDR SR--RRWPWR DLRD------ ---------- FVPLR---LV 
At26.5       -------EDK MNTEVSVSEK KSPRQNFPRR RGRKSLWRNT DDHGY----- FTPTLNEFFP 
Vv26.3       -------PSG EKTEVAVSEG DRKPKLFPRK QRKRSLWRNN RND------- FVPSLNELFP 
So17.4       ---------- ---------- --RLMDTLAL DSWVRNPFS- ---------I FGTAVAA-DA 
Sb17.4       ---------- ---------- --RLLDTLAL DSWVRNPFS- ---------I FGTAVAA-DA 
Zm17.4       ---------- ---------- --RLLDTLAL DSWVRNPFT- ---------I FGTAVAA-DA 
Cd16.6       ---------- ---------- --RLFDTLAL DTWK-NPFS- ---------I FGTPLSA-DA 
Hsp17.9B-CIX ---------- ---------- --KLFDTLAF DAWN--PFS- ---------I FGTTVAA-DA 
Zm16.6       ---------- ---------S SS---NPLSL DFWASSADP- ---------- FGVVRP---- 
So16.5       ---------- ---------R SSNVFDPLSL DFWTS-ADP- ---------- FGVVRP---- 
Hsp16.6-CVIII---------- ---------R SGNVLDPMSV DFWAD-ADP- ---------- FGAVRS---- 
Hsp16.9B-CI  ---------- ---------R RSNVFDPFSL DLWDPFDS-- ---------V FRSVVP--AT 
Hsp16.9A-CI  ---------- ---------R RSNVFDPFSL DLWDPFDS-- ---------V FRSVVP--AT 
Hsp16.9C-CI  ---------- ---------R RSNVFDPFA- DFWDPFDG-- ---------V FRSLVP--AT 
Zm17.2       ---------- ---------R RSNVFDPFSM DLWDPFDT-- ---------M FRSIVPS-AT 
Ta16.9B      ---------- ---------R RTNVFDPFAD LWADPFD--- ---------T FRSIVPA-IS 
Hsp17.9A-CI  ---------- ---------R RSNVFDPFSL DLWDPFDG-F PFGSGGSS-- SGSIFPSFPR 
Hsp18.0-CI   ---------- ---------R RSNVFDPFSL DLWDPFDG-F PFGSGSRS-- SGSIFPSFPR 
Hsp17.4-CI   ---------- ---------R RSNVFDPFSL DLWDPFDG-F PFGSG----- SGSLFP---R 
Hsp17.7-CI   ---------- ---------R RGNAFDPFSL DLWDPVDG-F PFGSGGSSSS SGSLFP---R 
Zm17.9       ---------- ---------R RGNAFDPFSL DLWDPFEGFF PFGSGGVR-- --SLVPSLPR 
At17.4CI     ---------- ----SFFGGR RTNVFDPFSL DVWDPFEG-- ---------- --FLTPGLTN 
Ta20.1       PQPQQHAAGH RANGHGHGGG GDEVMEPVSI DILEPFMEAI S--------- LTAFGGG--G 
Hv19.3       PH-QQQPAGH KTN--GHGGG G-EVVEPVSI DILEPFMEAI S--------- LKAFGG---- 
Ps19.8       QQPQQQAADH KAN--GHGGG GGEAVEPVSI DILEPFMEAI S--------- LTAFGAG--G 
Hsp18.8-CX   QNAQQKGGGG GGR---TGGG GGGEIEPVSV DIMEPFMDAI S--------- LTAFAAAPSA 
Vv16.4       ---------- ---------- ---------- SLFENLG--- ---------- ---------- 
Pt17.6       ---------- ---------- -----LLNQN SLFDPFRGF- ---------- ---------- 
Hsp17.8-CXI  ---------- ---------- -----RMLLD RFFPGAGGV- ---------- ---------- 
Zm22.7       AAP--VAALV PYGRPGG--- ----GLWDLM LLDDPFRVLE QS-------P LAASSVPRAS 
Hsp21.8-ER   MAPP-AAALV PYGYG----- ---------Y MLDDPFRVLE QS-------P LRPAGGVAAA 
Vv21.3       LIPIPAEGLM PYTRN----- ----LWDMVL PFDDPFRILE HS-------P ITVPKGLET- 
At22         NIKTS-EGSL SSALET--TP GSLLSDLWLD RFPDPFKILE RI-------P LGLERDTSVA 
Hsp23.2-ER   LASTAADGAL LPWFGGGGAR DEAVPELGLL AAADPFRILE HV-------P FGFDRDDVAM 
Hv17.1       ---------- ---------- -YPMQ---EV QQHPGFR-AV HPWQWWQWQ- -LSVLASSSS 
Hsp18.8-CV   ---------- ---------- -YPMEEEEEV HERPRFRRPV HPWQWHHWQN LLGLLSSSSP 
Zm17.2       ---------- ---------- -YPME----- EQHSGFR--- -QWQW-QWR- -LFSLLSSQP 
Vv15.7       ---------- ---------- ---------- -----FS--- -PFQPSSWH- --FLFTS--- 
At15.4       ---------- ---------- ---------- -----FQ--- -TIQVMPWE- --YVLAS--- 
Lp16.1CIII   ---------- ---------- --VVDVVSQL LFPESIERLV SP-----SRS ----NESKG- 
At17.4CIII   ---------- ---------- --AVAINHFF GLPEAIEKLI LPI----SRS GESNNESRGR 
Hsp18.6-CIII ---------- ---------- -FDTAVTSLL HLPEVLDRLG AAAGDRRSAG DHAHHAAHGH 
At17.6-CII   ---------- ---------- -RF----PII SILEDMLEVP EDHN----NE KTRNNPSRVY 
At17.7       ---------- ---------- -RF----PIF SILEDMLEAP EEQ-----TE KTRNNPSRAY 
Hsp18.0-CII  ---------- ---------- -MFGLETPLM TALQHLLDIP DGEGGAAGKQ GATGGPTRAY 
Hsp19.0-CII  ---------- ---------- -VVGGEPLLA VAMQQLLDLD LPDE----LE RQLNPPTRAY 
At15.7       ---------- ---------- -----MADRG IFLYPFRR-- ---------- ---------- 
Gm16.2       ---------- ---------- -----MAGT- IFGYPFRHFI WGH------- --------PP 
Hsp16.0-Px   ---------- ---------- -----MADL- FFGGPFRRIL YG-------- --------RP 
Vv22.4       ---------- ---------- PTVRKVFGDG SLFSPFLFRK FFDPS---DA FPLWEFESDV 
At21.7       ---------- ---------- GSEKAVFGEG SLFSPFLFGK YFDPS---DA FPLWEFEAEV 
Hsp22.3-CVI  ---------- ---------- AAARAVFGEG SLFSPFLFGK FFDPA---DA FPLWEFEPEV 
Zm21.9       ---------- ---------- TAARAVFAEG SLFCPFLFGN FFDPA---DP FPLWEFESDV 
Clustal Co                                                                     

             ....|....| ....|....| ....|....| ....|....| ....|....| ....|....| 
                125        135        145        155        165        175               
Le26.2       MSPMRTMRQM IDTMDRLFED -TMTFPGRNR A---SGTGEI RTPWDIHDDE NEIKMRFDMP 
Vv25.0       FSPMRTMRQM MDAMDRMFEE -TVAFPG--- ------SAEV RSPWDIVDDE NEIKMRFDMP 
At25.3       LSPMRTMRQM LDTMDRMFED -TMPVSGRNR GG--SGVSEI RAPWDIKEEE HEIKMRFDMP 
Hsp26.7-P    MSPMRTMRQM LDTMDRIFDD VALGFPATPR RSLATG--EV RMPWDVMEDD KEVRMRFDMP 
Ta26.7       MSPMRTMRQM LDTMDRLFDD -AVGFP-TAR RSLAAASEMP RMPWDIMEDD KEVKMRFDMP 
Zm26.4       MSPMRTMRQM LDTMDRLFDD -AVGFPMGTR RSPATTG-DV RLPWDIVEDE KEVKMRIDMP 
At23.6-M     FSPTRSVSQV LNLMDQFMEN ---PLLSA-T RGMGASGARR G--WDIKEKD DALYLRIDMP 
At23.5-M     FTPTRSLSQM LNFMDQVSEI ---PLVSA-T RGMGASGVRR G--WNVKEKD DALHLRIDMP 
Hsp26.2-MI   FGAPTSLGRL LALMEDAAVA ---TAAAPGT NGLATAAARR GGWWVAKEDD DAVHLKVSMP 
Hsp24.0-MI   FSAPQSLGRL LSLMDDLATP ---AGRAG-- ----AATLRR G--WNAKESE EALHLRVDMP 
Zm22.8       DGLGSALSQV AETLGRPLER ---------- ----L-APSR LLSGKVREDE ARYRLRFEVP 
Hsp23.6-MII  DGIGSALSQV AETLTRP--- ---------- ---------- -LTGKVREDE ERYRLRFEVP 
At26.5       PTIGNTLIQA TENMNRIFDN ---------- ----FNVNPF QLMGQVKEQD DCYKLRYEVP 
Vv26.3       PSIGNALMQA TQHMNRLLEN ---------- ----L--APS RLIGRLKEQD QCYKLRYEMP 
So17.4       WLASDTSAFA NTYIESRDT- ---------- ---------- ---------A GAYVFSAALP 
Sb17.4       WLASDTSAFA NTYIESRDT- ---------- ---------- ---------A GAYVFSAALP 
Zm17.4       WLASDTSAFA NTHIESRDT- ---------- ---------- ---------A AAYVFSAALP 
Cd16.6       WLASDTSAFA NTYIESRET- ---------- ---------- ---------A SAYVFSAALP 
Hsp17.9B-CIX WLASDTSAFA NTYIESRET- ---------- ---------- ---------A EAYVFRADLP 
Zm16.6       -LAEQCPVLT NVRVDWKET- ---------- ---------- ---------P EAHVFRADLP 
So16.5       -LAEQCPVLT NVRVDWKET- ---------- ---------- ---------P EAHVFRADLP 
Hsp16.6-CVIII-LAERCPVLT NVRVDWKET- ---------- ---------- ---------P TAHVFTADLP 
Hsp16.9B-CI  SDN-DTAAFA NARIDWKET- ---------- ---------- ---------P ESHVFKADLP 
Hsp16.9A-CI  SDN-DTAAFA NARIDWKET- ---------- ---------- ---------P ESHVFKADLP 
Hsp16.9C-CI  SDR-DTAAFA NARVDWKET- ---------- ---------- ---------P ESHVFKADLP 
Zm17.2       STNSETAAFA SARIDWKET- ---------- ---------- ---------P EAHVFKADLP 
Ta16.9B      GGGSETAAFA NARMDWKET- ---------- ---------- ---------P EAHVFKADLP 
Hsp17.9A-CI  GASSETAAFA GARIDWKET- ---------- ---------- ---------P EAHVFKADVP 
Hsp18.0-CI   GTSSETAAFA GARIDWKET- ---------- ---------- ---------P EAHVFKADVP 
Hsp17.4-CI   -ANSDAAAFA GARIDWKET- ---------- ---------- ---------P EAHVFKADVP 
Hsp17.7-CI   -ANSDAAAFA GARIDWKET- ---------- ---------- ---------P EVHVFKADVP 
Zm17.9       -TSSETAAFA GARIDWKET- ---------- ---------- ---------P EAHVFKADVP 
At17.4CI     APAKDVAAFT NAKVDWRET- ---------- ---------- ---------P EAHVFKADVP 
Ta20.1       CRPALGLPFS TASMDWKET- ---------- ---------- ---------P TAHVFMADVP 
Hv19.3       --PALGLPFS TASMDWKET- ---------- ---------- ---------P TAHVFMADVP 
Ps19.8       -GPALGMPFS TASMDWKET- ---------- ---------- ---------P TAHVFMADVP 
Hsp18.8-CX   AAAAAGVP-S TASMDWKET- ---------- ---------- ---------A AAHVFMADMP 
Vv16.4       -----IASSG YVHMDWKET- ---------- ---------- ---------P QAHIFQVDLP 
Pt17.6       -----LIENS ETQMDWKET- ---------- ---------- ---------P HAHVFEIDLP 
Hsp17.8-CXI  -----VAGEA RPPMDWRET- ---------- ---------- ---------P VAHVFEMDLP 
Zm22.7       LDSTSAAGVA LARCDWKET- ---------- ---------- ---------P DAHVISVDVP 
Hsp21.8-ER   AAAGEPAAVA LARCDWKET- ---------- ---------- ---------P EAHVVTVDVP 
Vv21.3       --------IA LARSDWKET- ---------- ---------- ---------T SAHIITLDVP 
At22         --------LS PARVDWKET- ---------- ---------- ---------A EGHEIMLDIP 
Hsp23.2-ER   --------LS MARVDWRET- ---------- ---------- ---------G DAHEVVVDVP 
Hv17.1       PPAPSTRPP- -NHVSWDET- ---------- ---------- ---------G AAHIYSADLP 
Hsp18.8-CV   SPATAAAAQR CSHVSWEET- ---------- ---------- ---------A AAHLYSASLP 
Zm17.2       ELLPPPRPA- -NHVRWEET- ---------- ---------- ---------A AAHLFSASLP 
Vv15.7       PLLVSYHFTP DNYVHWTET- ---------- ---------- ---------P ESHIYSANLP 
At15.4       QSLNNYQ--- ENHVRWSQS- ---------- ---------- ---------P DSHTFSVDLP 
Lp16.1CIII   ---------- TIPVDILDT- ---------- ---------- ---------P KEYIFYMDVP 
At17.4CIII   ------GSSN NIPIDILES- ---------- ---------- ---------P KEYIFYLDIP 
Hsp18.6-CIII GQHRISGIGG GAPVDIMET- ---------- ---------- ---------P GEYAFVLDVP 
At17.6-CII   MRDA--KAMA ATPADVIEH- ---------- ---------- ---------P NAYAFVVDMP 
At17.7       MRDA--KAMA ATPADVIEH- ---------- ---------- ---------P DAYVFAVDMP 
Hsp18.0-CII  VRDA--RAMA ATPADVKDL- ---------- ---------- ---------P GAYAFVVDMP 
Hsp19.0-CII  VRDR--RAMA NTPMDVKELR ---------- ---------- --------AS GALVLAVDMP 
At15.7       -FQEWSRS-- TALIDWMES- ---------- ---------- ---------N NSHIFKINVP 
Gm16.2       IFKEWSGS-- TALLDWLES- ---------- ---------- ---------P TAHILKINVP 
Hsp16.0-Px   FPPDWASASA TAAMDWVET- ---------- ---------- ---------P TSHVLRINVP 
Vv22.4       LLSHLRSS-G QTTVDWLQT- ---------- ---------- ---------D KDYVLKAELP 
At21.7       LLASLRSL-G QCRVDWSQT- ---------- ---------- ---------D QAYVLKSDIP 
Hsp22.3-CVI  LLAALRRG-A RTTVDWAET- ---------- ---------- ---------D SEYYLRADIP 
Zm21.9       LLAALRRGNA RTTVDWAET- ---------- ---------- ---------D CEYYLRADVP 
Clustal Co                                                                  :* 

             ....|....| ....|....| ....|....| ....|....| ....|....| ....|....| 
                185        195        205        215        225        235               
Le26.2       -GLSKEDVKV SVEND--MLV IK-------- ------GEHK ------KEED GR-------- 
Vv25.0       -GLSKEDVKV SVEDD--LLV IK-------- ------GEQK ------KEEG EK-------- 
At25.3       -GLSKEDVKI SVEDN--VLV IK-------- ------GEQK ------KEDS D--------- 
Hsp26.7-P    -GLSREEVKV MVEDD--ALV IR-------- ------GEHK ------KEEG EG---AEGSG 
Ta26.7       -GLSREEVKV MVEGD--ALV IR-------- ------GEHK ------KEAG EGQGEAAEGG 
Zm26.4       -GLARDEVKV MVEDD--TLV IR-------- ------GEHK ------KEEG AEGG-SGGDG 
At23.6-M     -GLSREDVKL ALEQD--TLV IR-------- ------GEGK ------NEED G--------- 
At23.5-M     -GLSREDVKL ALEQN--TLV IR-------- ------GEGE ------TEE- ---------- 
Hsp26.2-MI   -GLGKEHVKV WAEQN--SLV IK-------- ------GEGE ------KDP- ---------- 
Hsp24.0-MI   -GLGKEHVKV WAEQN--SLV IK-------- ------GEGE ------KEA- ---------- 
Zm22.8       -GLGKGDVRV AVEDG--VLV IE-------- ------GEKR EH----GEEG DG-------- 
Hsp23.6-MII  -GLGKDDVRV YVDDG--VLA IH-------- ------GEKR DV----VEED RGRD----GD 
At26.5       -GLTKEDVKI TVNDG--ILT IK-------- ------GDHK ------AEEE KGSP----EE 
Vv26.3       -GLTKEDVKI SVEDG--ILS IR-------- ------GEHK ------EEEE EGS------D 
So17.4       PGVKKEEVTV EVDEG-NVLV IT-------- ------GERS V-----SREE RVG-DRWHHV 
Sb17.4       PGVRKEEVTV EVDEG-NVLV IT-------- ------GQRS V-----SREE RVG-DRWHHV 
Zm17.4       PGVKKEEVTV ELDEG-NVLV IA-------- ------GERS V-----CRQE RS--DGCHHI 
Cd16.6       PGVKKEEVRV EVDED-NVLE IT-------- ------GERS V-----RREE KG--DKWHHI 
Hsp17.9B-CIX AGVKKEEVRV EVDEG-NVLV IT-------- ------GERS V-----RREE KG--QRSHHI 
Zm16.6       -GVRKEAAKV EVEDG-NVLV IS-------- ------GERA RE---EEEAG KDEAWRWRLV 
So16.5       -GVKKEAAKV EVEDG-NVLV IS-------- ------GERA R-----EEAG KDE--KWRLV 
Hsp16.6-CVIII-GVRKDQAKV EVEDG-GVLV IS-------- ------GERA RE---EDVDG KNDE-RWHHV 
Hsp16.9B-CI  -GVKKEEVKV EVEEG-NVLV IS-------- ------GQRS K-----EKED KND--KWHRV 
Hsp16.9A-CI  -GVKKEEVKV EVEEG-NVLV IS-------- ------GQRS K-----EKED KND--KWHRV 
Hsp16.9C-CI  -GVKKEEVKV EVEEG-NVLV IS-------- ------GQRS K-----EKED KND--KWHRV 
Zm17.2       -GVKKEEVKV EVEDG-NVLV IS-------- ------GQRS R-----EKED KDD--KWHRV 
Ta16.9B      -GVKKEEVKV EVEDG-NVLV VS-------- ------GERT K-----EKED KND--KWHRV 
Hsp17.9A-CI  -GLKKEEVKV EVDDG-NILQ IS-------- ------GERN K-----EQEE KTD--QWHRV 
Hsp18.0-CI   -GLKKEEVKV EVEDG-NVLQ IS-------- ------GERS K-----EQEE KTD--KWHRV 
Hsp17.4-CI   -GLKKEEVKV EVEDG-NVLQ IS-------- ------GERI K-----EQEE KTD--KWHRV 
Hsp17.7-CI   -GLKKEEVKV EVDDG-NILQ IS-------- ------GERS R-----EQEE KSD--KWHRV 
Zm17.9       -GLRKEEVKV EVEDG-NVLQ IS-------- ------GERN K-----EHEE KND--RWHRV 
At17.4CI     -GLKKEEVKV EVEDG-NILQ IS-------- ------GERS S-----ENEE KSD--TWHRV 
Ta20.1       -GLRREEVKV EVEQE-RVLR IS-------- ------GQRA R-----AAED KGD--RWHRV 
Hv19.3       -GLRREEVKV EVEQE-RVLR IS-------- ------GQRA R-----AAED KGD--RWHRV 
Ps19.8       -GLRREEVKV EVEQE-RVLR IS-------- ------GQRA R-----RAED KGD--RWHRV 
Hsp18.8-CX   -GVRREEVRV EVEEE-KVLR IS-------- ------GQRA R-----AAEE KGE--RWHRV 
Vv16.4       -GLTKNEVKL EVHQG-RVLH I--------- ------SGC- RE---EEPEE KGE--KWHCR 
Pt17.6       -GLTKEDVKI EVHEG-TVLQ I--------- ------STAE RK---EEAEE KGD--KWHCK 
Hsp17.8-CXI  -GLAKDQVAV EVVDG-HILR VRAGGEHEDA NNAAKAGKAS GE---EEEEN DGV--RWHCR 
Zm22.7       -GVRREDVKV EVEENSRVLR VS-------- ------GERR ---ADEEKEG DR----WHXA 
Hsp21.8-ER   -GVRRGDVRV EVDEASRVLR VS-------- ------GERR RAGAAEEEEG ERDGVRWHRA 
Vv21.3       -GMKKEDIKI EIEEN-RVLR IS-------- ------GERT ---AEGEAEG EK----WHRS 
At22         -GLKKDEVKI EVEEN-GVLR VS-------- ------GERK ---REE--EK KG--DQWHRV 
Hsp23.2-ER   -GMRKEDLRV EVEDN-RVLR IS-------- ------GERR ---REETTEQ KGGGDHWHRE 
Hv17.1       -GVKKEEIRV EIEDG-RYLV VRT------- ---------- ------ELDA G---DAEVHG 
Hsp18.8-CV   -GVRKEEIRV EVEDA-MYLV IRT------- ---------- ------ELDD GGDGDGGGGG 
Zm17.2       -GVRKEEIRV EVEDA-RYLV IRT------- ---------- ------ELDA G-TGTGGAVA 
Vv15.7       -GVRKEEIRV ELEDS-RYLI IRT------- ---------- ------EAID ES------TK 
At15.4       -GLRKEEIKV EIEDS-IYLI IRT------- ---------- ------EATP MSP----PDQ 
Lp16.1CIII   -GLSKSDLQV SVEDE-KTLV IRS------- -----NGKRK R-----EESE -EEGCKYVRL 
At17.4CIII   -GISKSDIQV TVEEE-RTLV IKS------- -----NGKRK R-----DDDE SEEGSKYIRL 
Hsp18.6-CIII -GLSKSDIQV TLEED-RVLV MKSSNG---- ---AGNGKRK R-----EEEE --GECKYIRL 
At17.6-CII   -GIKGDEIKV QVEND-NVLV VSG------- -------ERQ R-----ENKE N-EGVKYVRM 
At17.7       -GIKGDEIQV QIENE-NVLV VSG------- -------KRQ R-----DNKE N-EGVKFVRM 
Hsp18.0-CII  -GLKSSDIKV QVEEE-RLLV ISG------- -------ERR RG--GGEEEK E-ESCKYLRM 
Hsp19.0-CII  -GVAPADVRV EVEDG-NVLA ISG------- -------ERR RP--AGDGDD GGEGVKYLRM 
At15.7       -GYNKEDIKV QIEEG-NVLS IR-------- ------GEG- ---IKEE--- KKENLVWHVA 
Gm16.2       -GFSKEDIKV QIEDG-NILH IK-------- ------GEV- ---WREEPQA KEKDTVWHVA 
Hsp16.0-Px   -GLGKDDVKV QVEDG-NVLT VR-------- ------GAAP HA-AAEKERE REKDVVWHVA 
Vv22.4       -GVGKNSVQV YVE-NGKVVE ISG------- -----LWRHQ K------EPK T-KEWRSGHW 
At21.7       -VVGKNNVQV YVDINGRVME ISG------- -----QWNSN K------KAA TNSDWRSGRW 
Hsp22.3-CVI  -GGRKCDVEV SGDDAMRVVD VSG------- -----LWRAA PP----PPPP DGRDWRAGRW 
Zm21.9       -GGRRCDVEV SG-DAMKVID ICG------- -----LWRA- -------PAA DGRDWRCGRW 
Clustal Co            :         :  :                                           

             ....|....| ....|....| ....|....| ....|....| ....|....| ....|....| 
                245        255        265        275        285        295               
Le26.2       DKHSWGRNYS SYDTRLSLPD NVVK-DK-IK AELKN----G VLFISIPKTE VE-------- 
Vv25.0       DSWS-GSGFS SYSTRLQLPD NCEK-DK-IK AELKN----G VLSISIPKTK VE-------- 
At25.3       DSWS-GRSVS SYGTRLQLPD NCEK-DK-IK AELKN----G VLFITIPKTK VE-------- 
Hsp26.7-P    DGWWKERSVS SYDMRLALPD ECDK-SK-VR AELKN----G VLLVTVPKTE VE-------- 
Ta26.7       DGWWKERSVS SYDMRLALPD ECDK-SQ-VR AELKN----G VLLVSVPKRE TE-------- 
Zm26.4       DGWWKQRSVS SYDMRLALPD ECDK-SK-VR AELKN----G VLLVTVPKTE VE-------- 
At23.6-M     -GEEGESGNR RFTSRIGLPD KIYKIDE-IK AEMKN----G VLKVVIPKMK EQ-------- 
At23.5-M     -GEDVSGDGR RFTSRIELPE KVYKTDE-IK AEMKN----G VLKVVIPKIK ED-------- 
Hsp26.2-MI   -EDDADAAPP RYTRRIELPA DAFKMDK-IK AEMKN----G VLRVAVPKLK EE-------- 
Hsp24.0-MI   -GEDEGAAPA RYSGRIELAP EVYRMDQ-IK AEMKN----G VLKVVVPKVK EE-------- 
Zm22.8       GEWWSTSGY- --HASLLLPD DARA-EG-IT AEVKD----G VLYVTVPRTG -E-------- 
Hsp23.6-MII  GECWAAATY- --HAGLLLPE DAVA-EG-IT AEVRD----G VLHVTVPRSP -E-------- 
At26.5       DEYWSSKSYG YYNTSLSLPD DAKV-ED-IK AELKN----G VLNLVIPRTE -K-------- 
Vv26.3       DEHWSATSYG YYDTSLLLPT DAKI-EE-IK AELKD----G VLTIIIPRNE -K-------- 
So17.4       ERCC-----A SFLGRFHLPD DAAVNG--VR AAMDA----G MLTVTVPKVG ASR--DCGGG 
Sb17.4       ERCC-----A SFLGRFHLPE DAAVDG--VR AAMDA----G MLTVTVPKVG AA----AAAI 
Zm17.4       ERSR-----A TFLARFHLPE DAAVDG--VR AALDA----G RLTVTVPKVG AA---AAAIV 
Cd16.6       ERSS-----A TFLGRFHLPE DAVVDG--VR AAMDG----G MLTVTVPKVG AA-------- 
Hsp17.9B-CIX ERSC-----A TFFGRFHLPD DAVVDL--VR ASMDG----G MLTVTVPKVV TDKQPAIAAA 
Zm16.6       ERSS-----G RFQRRFRLPR GARLDQ--VH ASMEN----G VLTVTVPKEE AK-------- 
So16.5       ERSC-----G RFQRRFRLPR GAKLDQ--VR ASMDN----G VLTVTVPKEE VK-------- 
Hsp16.6-CVIIIERSS-----G KFQRRFRLPR GARVDQ--VS ASMDN----G VLTVTVPKEE TK-------- 
Hsp16.9B-CI  ERSS-----G QFMRRFRLPE NAKVDQ--VK AGMEN----G VLTVTVPKAE VK-------- 
Hsp16.9A-CI  ERSS-----G QFMRRFRLPE NAKVDQ--VK AGLEN----G VLTVTVPKAE VK-------- 
Hsp16.9C-CI  ERSS-----G QFMRRFRLPE NAKVDQ--VK ASMEN----G VLTVTVPKAE VK-------- 
Zm17.2       ERSS-----G QFIRRFRLPD DAKVDQ--VK AGLEN----G VLTVTVPKAE EK-------- 
Ta16.9B      ERSS-----G KFVRRFRLLE DAKVEE--VK AGLEN----G VLTVTVPKAE VK-------- 
Hsp17.9A-CI  ERSS-----G KFLRRFRLPD NAKPEQ--IK ASMEN----G VLTVTVPKEE AK-------- 
Hsp18.0-CI   ERSS-----G KFLRRFRLPE NTKPEQ--IK ASMEN----G VLTVTVPKEE PK-------- 
Hsp17.4-CI   ERSS-----G KFLRRFRLPE NTKPEQ--IK ASMEN----G VLTVTVPKEE PK-------- 
Hsp17.7-CI   ERSS-----G KFLRRFRLPE NTKPEQ--IK ASMEN----G VLTVTVPKEE PK-------- 
Zm17.9       ERSS-----G KFLRRFRLPD NAKADQ--IK ASMEN----G VLTVTVPKEE AK-------- 
At17.4CI     ERSS-----G KFMRRFRLPE NAKVEE--VK ASMEN----G VLSVTVPKVQ ES-------- 
Ta20.1       ERSA-----E KFVRTVRLPP NADVDGGGVH AALDN----G VLTITIPKDD GK-------- 
Hv19.3       ERSA-----E KFVRTVRLPP NADVDGGGVH AALDN----G VLTITIPKDD GK-------- 
Ps19.8       ERSA-----E KFVRTVRLPP NADVDGGGVH AALDN----G VLTITIPKDD GK-------- 
Hsp18.8-CX   ERSS-----E RFVRTVRLPP NANTDG--VH AALDN----G VLTITIPKDN DR-------- 
Vv16.4       ERSC-----G SFSRQFRLPE DAKVEE--IK ASMHD----G VLIVTVPKDE AL-------- 
Pt17.6       ERSR-----G GFSRRFRLPE NAKLDE--IK ASMHD----G VLVVTVPKDE LK-------- 
Hsp17.8-CXI  ERAA-----G ---------R RRAADE--AS ARMAD----G VLTVTVPKRK GK-------- 
Zm22.7       ERAA-----G RFWRRFRMPA GADVDR--VS ARLEN----G VLTVTVPKVA GH-------- 
Hsp21.8-ER   ERAA-----G RFWRRFRMPP GADVGR--VA ARLDD----G VLTVTVPKVP GH-------- 
Vv21.3       ERAT-----G KFWRQFRLPA NADLDR--IK AHLEN----G VLRITIPKLA ED-------- 
At22         ERSY-----G KFWRQFKLPD NVDMES--VK AKLEN----G VLTINLTKLS PE-------- 
Hsp23.2-ER   ERSY-----G RFWRQLRLPD NADLDS--IA ASLDN----G VLTVRFRKLA PD-------- 
Hv17.1       RRGG------ -FARKFRLPG MVDADG--IT AEYAH----G VLTVTVPRMH NR-------- 
Hsp18.8-CV   GRRS------ -FARKFRLPA MVDADG--IS AEYTH----G VLRVTVPRLH TR-------- 
Zm17.2       DARS------ -FDRKFRLPG MVDVDG--IS AAYTH----G VLTVKVPRMH TR-------- 
Vv15.7       PAKS------ -FMRKFRLPD MIDIDG--IS AGYED----G VLTVTVPRSF VR-------- 
At15.4       PLKT------ -FKRKFRLPE SIDMIG--IS AGYED----G VLTVIVPKRI MT-------- 
Lp16.1CIII   ERNP----PL KLMRKFKLPD YCNVSA--IT AKCEN----G VLTVVVEKMP P--------- 
At17.4CIII   ERRL----AQ NLVKKFRLPE DADMAS--VT AKYQE----G VLTVVIKKLP PQ-------- 
Hsp18.6-CIII ERRAS---PR AFARKFRLPE DADTGG--IS ARCEN----G VLTVTVKKRP PP-------- 
At17.6-CII   ERRM-----G KFMRKFQLPE NADLDK--IS AVCHD----G VLKVTVQKLP PP-------- 
At17.7       ERRM-----G KFMRKFQLPD NADLEK--IS AACND----G VLKVTIPKLP PP-------- 
Hsp18.0-CII  ERRM-----G KFMRKFVLPD NADVDK--IS AVCQD----G VLTVTVEKLP PP-------- 
Hsp19.0-CII  ERRM-----G KFMRRFPLPE SADLDG--VR AEYKD----G VLTVTVDKKP PP-------- 
At15.7       EREAFSGGGS EFLRRIELPE NVKVDQ--VK AYVEN----G VLTVVVPKDT SS-------- 
Gm16.2       ERGTGKGG-- -FSREIELPE NVKVDQ--IK AQVEN----G VLTIVVPKDA TP-------- 
Hsp16.0-Px   ERGR-----P EFAREVALPA EVRVEQ--IR ASVDN----G VLTVVVPKEP AP-------- 
Vv22.4       WEHG------ -YVRRLELPE NADWRR--IE ASVK---DEI YLEIRIPKCD IP-------- 
At21.7       WEHG------ -YVRRLELPS DADAKY--SE AFLSNNDDYS FLEIRIPKIN SK-------- 
Hsp22.3-CVI  WEHG------ -FVRRVELPE DADWRK--VE AFFD--DGEG LLEIKVPKSG DA-------- 
Zm21.9       WEHG------ -FVRRVELPE DAEWRK--VE AHFD--DCEG ALEIKVPKTA DD-------- 
Clustal Co                                    *           * : . :              

             ....|....| ....|....| ....|....| ....|....| ..
                305        315        325        335             
Le26.2       ---KKVIDVQ IN-------- ---------- ---------- --
Vv25.0       ---RKVIDVQ IQ-------- ---------- ---------- --
At25.3       ---RKVIDVQ IQ-------- ---------- ---------- --
Hsp26.7-P    ---RKVIDVQ VQ-------- ---------- ---------- --
Ta26.7       ---RKVIDVK VQ-------- ---------- ---------- --
Zm26.4       ---RKVIDVQ VQ-------- ---------- ---------- --
At23.6-M     ---ERNDVRQ IEIN------ ---------- ---------- --
At23.5-M     ---ERNNIRH INVD------ ---------- ---------- --
Hsp26.2-MI   ---ERKDVFQ VNVE------ ---------- ---------- --
Hsp24.0-MI   ---QRRDVFQ VNVE------ ---------- ---------- --
Zm22.8       ---RKRNVTE VKVQ------ ---------- ---------- --
Hsp23.6-MII  ---RKRSVTE VKVR------ ---------- ---------- --
At26.5       ---PKKNVQE ISVE------ ---------- ---------- --
Vv26.3       ---KGKDVKE VQIQ------ ---------- ---------- --
So17.4       GEKPN-EGPA PLVS---GPA CC-------- ---------- --
Sb17.4       AEKPN-EVPA AVEAADGGPA C--------- ---------- --
Zm17.4       PEKPAADAPA LAPVPVEAGP C--------- ---------- --
Cd16.6       AEMPE--AKA AIEA---GP- ---------- ---------- --
Hsp17.9B-CIX APVPAVVAPA VEAKAIEASP ---------- ---------- --
Zm16.6       --KPQ--VRA VEISG----- ---------- ---------- --
So16.5       --KPQ--VRA VEISG----- ---------- ---------- --
Hsp16.6-CVIII--KPQ--LKA IPISG----- ---------- ---------- --
Hsp16.9B-CI  --KPE--VKA IEISG----- ---------- ---------- --
Hsp16.9A-CI  --KPE--VKA IEISG----- ---------- ---------- --
Hsp16.9C-CI  --KPE--VKA IEISG----- ---------- ---------- --
Zm17.2       --KPE--VKA IEISG----- ---------- ---------- --
Ta16.9B      --KPE--VKA IQISG----- ---------- ---------- --
Hsp17.9A-CI  --KPD--VKS IQISG----- ---------- ---------- --
Hsp18.0-CI   --KPD--VKS IQVTG----- ---------- ---------- --
Hsp17.4-CI   --KPD--VKS IQITG----- ---------- ---------- --
Hsp17.7-CI   --KPD--VKS IQISG----- ---------- ---------- --
Zm17.9       --KAD--VKN VQITG----- ---------- ---------- --
At17.4CI     --KPE--VKS VDISG----- ---------- ---------- --
Ta20.1       --KAY--GRI IPITN----- ---------- ---------- --
Hv19.3       --KAY--GRI IPITN----- ---------- ---------- --
Ps19.8       --KAY--GRN IPITN----- ---------- ---------- --
Hsp18.8-CX   --KPH--ARI IPITN----- ---------- ---------- --
Vv16.4       --MKHSQKNM VEISGDD-EA HAPKGLGRFV CCKA------ --
Pt17.6       --TKPKNK-A VEISGDDGEK HVSRGLGRFV CCKA------ --
Hsp17.8-CXI  --KRHAGN-- GKAAGDD--- -------KPV CCRFWP---- --
Zm22.7       ---RGREPRV ISIAGGD--M GGVDVA---- ---EVMASKA EM
Hsp21.8-ER   ---RGREPRV VAIDGAG--A GDMEAE---- ---VVKASKA EM
Vv21.3       ---RKKQAKV VNIAEET--N SGED------ ----VMATKS EM
At22         ---KVKGPRV VNIAAEE-DQ TAKISS---- ----SESKEL --
Hsp23.2-ER   ---QIKGPRV VGIASAGGDD GGKKSIGGAG EGQNQQAKKV EL
Hv17.1       ---ARPMVNL IGSG-----P ACDPVARAA- ---------- --
Hsp18.8-CV   ---ARPVVNL AGGGGGGGGP ACDPVARAA- ---------- --
Zm17.2       ---ARPVVDI LGAG---AGP ASDHAARAA- ---------- --
Vv15.7       ---RGFYIDP GDLP-----E QLELLARAA- ---------- --
At15.4       ---R-RLIDP SDVP-----E SLQLLARAA- ---------- --
Lp16.1CIII   ----PSKAKT VKVA------ ----VS---- ---------- --
At17.4CIII   ----PPKPKT VQIA------ ----VS---- ---------- --
Hsp18.6-CIII ----EKKTKS VQVT------ ----IA---- ---------- --
At17.6-CII   ---EPKKPKT IQVQ------ ----VA---- ---------- --
At17.7       ---EPKKPKT IQVQ------ ----VA---- ---------- --
Hsp18.0-CII  ---EPKKPKT IEVK------ ----VA---- ---------- --
Hsp19.0-CII  ---EPKKPRV VEVK------ ----VAGAGE PKGKGK---- --
At15.7       ---KSSKVRN VNITSKL--- ---------- ---------- --
Gm16.2       ---KTPKVRN INITSRL--- ---------- ---------- --
Hsp16.0-Px   ---ARPRTRP IAVSSKL--- ---------- ---------- --
Vv22.4       ---HGKEEGA EDSE------ ---------- ---------- --
At21.7       ---NKF---- ---------- ---------- ---------- --
Hsp22.3-CVI  ---HQAAAAT A--------- ---------- ---------- --
Zm21.9       ---DDAHHAT A--------- ---------- ---------- --
Clustal Co                                                 
