# Supplementary material for: Rice sHsp genes: genomic organization and expression profiling under stress and development
Source: BMC Genomics. 2009 Aug 24;10:393. doi: 10.1186/1471-2164-10-393 (PMC2746236; doi:10.1186/1471-2164-10-393)
Supplement: Additional file 2 — Supplemental Table 1. Predicted cellular location of organellar sHsps of rice. Supplemental Table 2. List of primer sets for RT- PCR. [file 1471-2164-10-393-S2.doc]

| **Supplemental Table 1. Predicted cellular location of organellar sHsps of rice.** | | | | |  |
| --- | --- | --- | --- | --- | --- |
|  |  |  |  |  |  |
| **Protein** | **Psort** | **Predotar** | **TargetP** | **Localization** | **Transit peptide** |
|  |  |  |  |  |  |
| Hsp23.6-MII | outside (0.370) | MT (0.63) | MT (RC2) 0.822 | MT | 52 |
| Hsp26.2-MI | MT (0.661) | MT (0.71) | MT (RC4) 0.490 | MT | 33 |
| Hsp24.0-MI | MT (0.672) | MT (0.23) | MT (RC5) 0.334 | MT | 35 |
| Hsp26.7-P | MT (0.737) | CP (0.67) | MT (RC5) 0.598 | CP/MT | 49 |
| Hsp23.2-ER | outside 0.820) | ER (0.98) | SP (RC1) 0.993 | ER | 27 |
| Hsp21.8-ER | outside 0.820) | ER (0.66) | SP (RC2) 0.801 | ER | 29 |
| Hsp16.0-Px | Px (0.809) | none (0.88) | other 0.377 | Px | none |
| For cellular localization prediction, each protein was analyzed with Psort, Predotar and Target P. Localization (CP- chloroplast, ER- endoplasmic reticulum, MT- mitochondrion,SP-secretory pathway, Px- peroxisome) is followed by probability score in each prediction algorithm. TargetP predictions are followed by reliability class (RC) which indicates the confidence in prediction, RC1= highest and RC5 = lowest. The cleavage site for transit peptide for mitochondrial and chloroplastic sHsps was predicted by MitoProt and ChloroP, respectively. Signal peptide for secretory pathway was predicted by TargetP. | | | | | |

| **Supplemental Table 2. List of primer sets for RT- PCR** | | |  |
| --- | --- | --- | --- |
|  |  |  |  |
| **Gene** | **Forward Sequence (5'→3')** | **Reverse Sequence (5'→3')** | **AT** |
|  |  |  |  |
| Hsp16.9A-CI | TGAAGATGTGATCGGTGAGG | GTTTGCAACCTGAAAAGCTC | 55°C |
|  |  |  |  |
| Hsp17.9A-CI | ATGTCGCTGATCCGCCGCAG | AGGAGGCGCCGCGCGGGAAG | 58°C |
|  |  |  |  |
| Hsp17.4-CI | GCATTGGGCTAATCTAAAAC | AGTAGTAGAACATAATATAG | 50°C |
|  |  |  |  |
| Hsp18.8-CX | GAGCATGGACTGGAAGGAGA | GTGAGGCTTCCTGTCGTTGT | 55°C |
|  |  |  |  |
| Hsp23.6-MII | GAGGTGAGGGACGGCGTGCT | CAAAATTCATCAAGACTATG | 48°C |
|  |  |  |  |
| Hsp26.2-MI | ACCAACGGCCTCGCAACCGC | CTACTCGACATTGACCTGGA | 56°C |
|  |  |  |  |
| Hsp24.0-MI | GGTCAACGTCGAGTAGGAGC | AAGGACGCCTTGGCTTTAAT | 56°C |
|  |  |  |  |
| Hsp26.7-P | CGATGCGGACGATGCGGCAG | TCCAGAGTGATCGTTCAGTA | 55°C |
|  |  |  |  |
| Hsp23.2-ER | GTGGCAAGAAGAGCATCGGC | CACAACGATGAAAGTGCGAT | 56°C |
|  |  |  |  |
| Hsp21.8-ER | GACGTGCGCGTGGAGGTGGA | TCGCCGATCACTATTCACTG | 55°C |
|  |  |  |  |
| Hsp18.6-CIII | TCACCATCGCCTGAATCATA | TAATTTGCTTGATCCTCACA | 48°C |
|  |  |  |  |
| Hsp16.0-Px | CTCCAGCAAGCTCTGATCCT | GCAGAAGACCCACCTTTATT | 56°C |
|  |  |  |  |
| Hsp18.0-CII | AGGAGGAGAGGCTGCTGGTG | ATCACATCGCATACGGCATA | 54°C |
|  |  |  |  |
| Hsp19.0-CII | ATGGGCAAGTTCATGAGGAG | TCCAGGACCATCATGATGAT | 52°C |
|  |  |  |  |
| Hsp17.8-CXI | ATGTCTTTGGTGCTCTCGCG | TCACGGCCAGAAGCGGCAGC | 56°C |
|  |  |  |  |
| Hsp22.3-CVI | TGAGAATCCTAACCTCGCCG | GAATCGAATTTATGATCTCG | 50°C |
|  |  |  |  |
| Hsp18.8-CV | CCTGATTAGTGATTCGATAT | TCCTTCACGTTTTACTGCGA | 50°C |
|  |  |  |  |
| Acd18.0 | CACCCTCACCATCGATCTCT | ATCAGTGCTCGTCATCTCA | 50°C |
|  |  |  |  |
| Acd21.0 | ATTTCACCCGTTGTTCTGGT | CTACTTGACAGAGATATCGA | 48°C |
|  |  |  |  |
| Acd38.4 | AGAAATGTGCCAACGAAGGA | TACATAAACATATCTTCTCT | 50°C |
|  |  |  |  |
| Acd50.4 | AGGAAAAGGACACCGACCTT | AACAGGCCCAAGTCAATCAC | 52°C |
|  |  |  |  |
| Ubq 5 | GCACAAGCACAAGAAGGTGA | TCGATTTCCTCCTCCTTCCT | 52°C |
| PCR was performed for 25 cycles for all the genes except *Hsp26.2*-MI, which was subjected to 30 cycles of amplification. AT denotes annealing temperature. | | |  |
