# Supplementary material for: Rice sHsp genes: genomic organization and expression profiling under stress and development
Source: BMC Genomics. 2009 Aug 24;10:393. doi: 10.1186/1471-2164-10-393 (PMC2746236; doi:10.1186/1471-2164-10-393)
Supplement: Additional file 3 — Amino acid sequence alignment of new subfamilies. [file 1471-2164-10-393-S3.pdf]

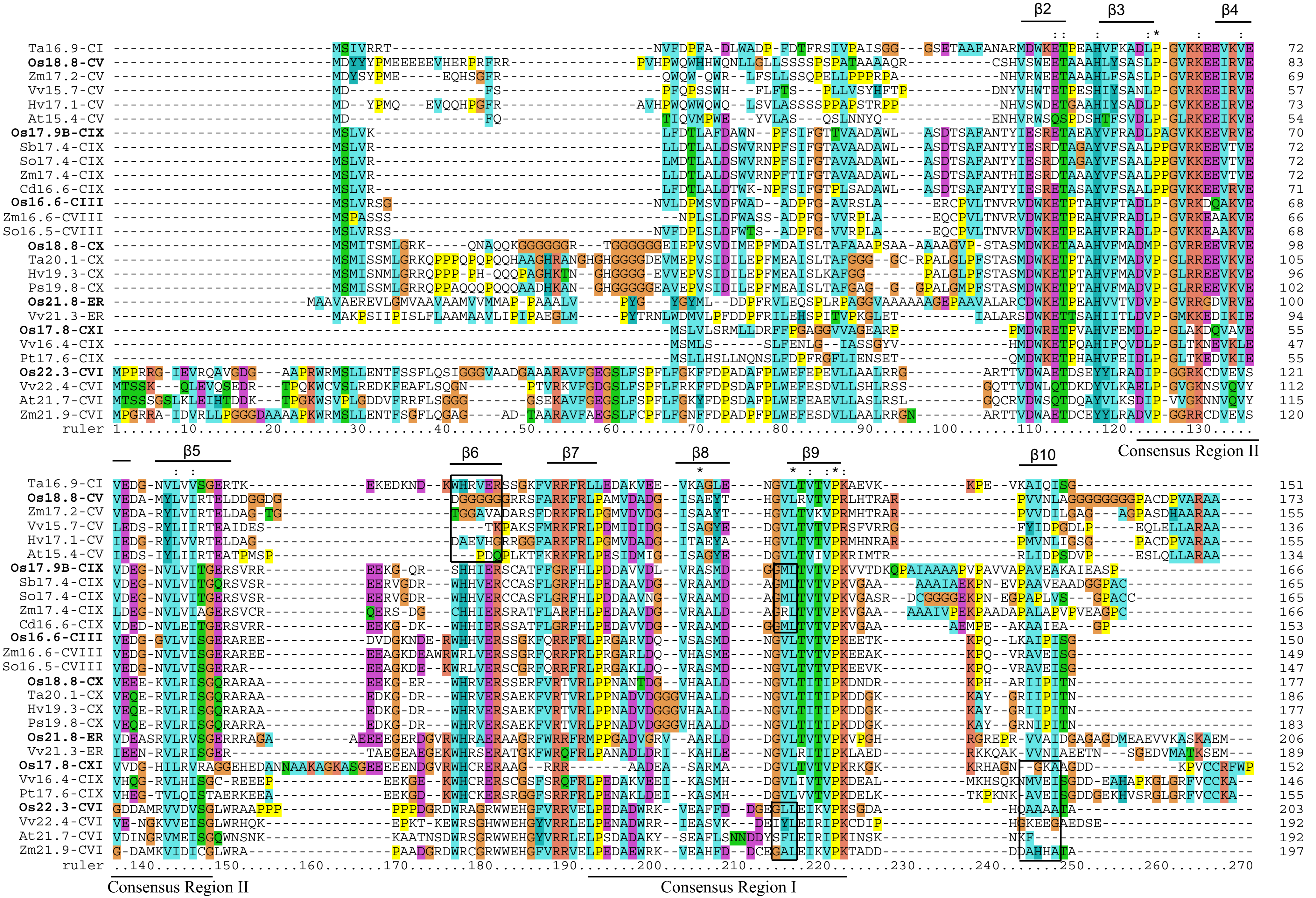

Supplemental Figure 1. Amino acid sequence alignment of recent and new subfamilies. The amino acid sequences of sHsps were aligned with wheat Hsp16.9-CI by ClustalX1.83. The predicted β-strands depicted by thick lines above the alignment are based on their position in known structure of wheat Hsp16.9 [van Montfort RL, Basha E, Friedrich KL, Slingsby C, Vierling E: Crystal structure and assembly of a eukaryotic small heat shock protein. Nat Struct Biol 2001, 8(12):1025-1030]. Black box in the consensus region I and β10-strand depicts unconserved regions in CVI and CIX subfamilies. The accession numbers of sHsps are given in Fig. 2.
