# Supplementary material for: Rice sHsp genes: genomic organization and expression profiling under stress and development
Source: BMC Genomics. 2009 Aug 24;10:393. doi: 10.1186/1471-2164-10-393 (PMC2746236; doi:10.1186/1471-2164-10-393)
Supplement: Additional file 4 — Supplemental Figure 2. Expression pattern of sHsps during anther development in rice. [file 1471-2164-10-393-S4.ppt]

## Slide 1
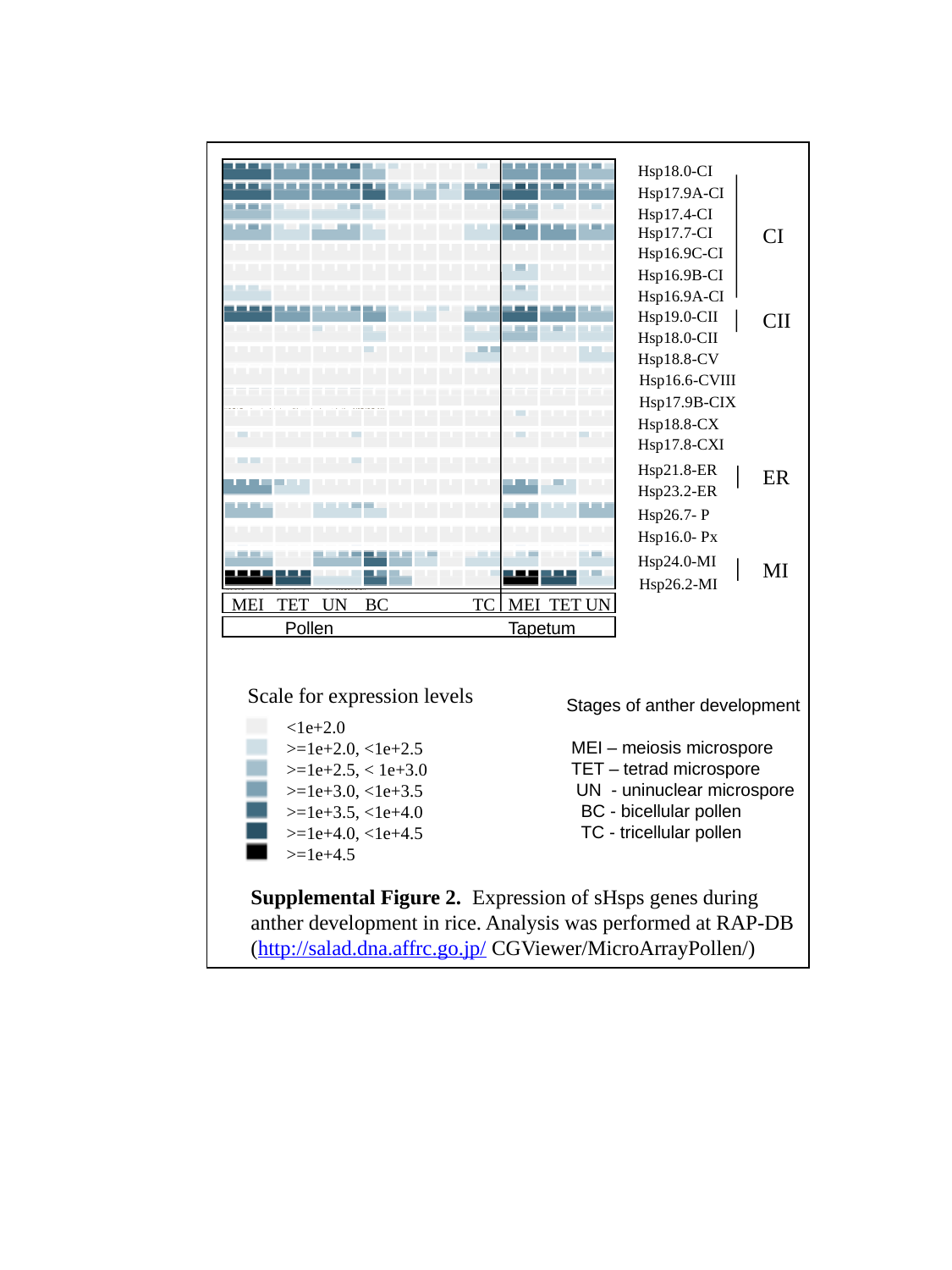

Hsp18.0-CI
 MEI TET UN BC TC MEI TET UN
 Pollen Tapetum
Hsp17.9A-CI
Hsp17.4-CI
CI
Hsp17.7-CI
Hsp16.9C-CI
Hsp16.9B-CI
Hsp16.9A-CI
Hsp19.0-CII
CII
Hsp18.0-CII
Hsp18.8-CV
Hsp16.6-CVIII
Hsp17.9B-CIX
Hsp18.8-CX
Hsp17.8-CXI
Hsp21.8-ER
ER
Hsp23.2-ER
Hsp26.7- P
Hsp16.0- Px
Hsp24.0-MI
MI
Hsp26.2-MI
Scale for expression levels
Stages of anther development
 MEI – meiosis microspore
 TET – tetrad microspore
 UN - uninuclear microspore
 BC - bicellular pollen
 TC - tricellular pollen
<1e+2.0
>=1e+2.0, <1e+2.5
>=1e+2.5, < 1e+3.0
>=1e+3.0, <1e+3.5
>=1e+3.5, <1e+4.0
>=1e+4.0, <1e+4.5
>=1e+4.5
Supplemental Figure 2. Expression of sHsps genes during anther development in rice. Analysis was performed at RAP-DB (http://salad.dna.affrc.go.jp/ CGViewer/MicroArrayPollen/)
