# Supplementary material for: Rice sHsp genes: genomic organization and expression profiling under stress and development
Source: BMC Genomics. 2009 Aug 24;10:393. doi: 10.1186/1471-2164-10-393 (PMC2746236; doi:10.1186/1471-2164-10-393)
Supplement: Additional file 5 — Supplemental figure 3(A). Expression pattern of Acd genes during development stages of rice plant. Supplemental figure 3(B). Microarray based expression profiles of Acd genes of rice under heat stress. [file 1471-2164-10-393-S5.pdf]

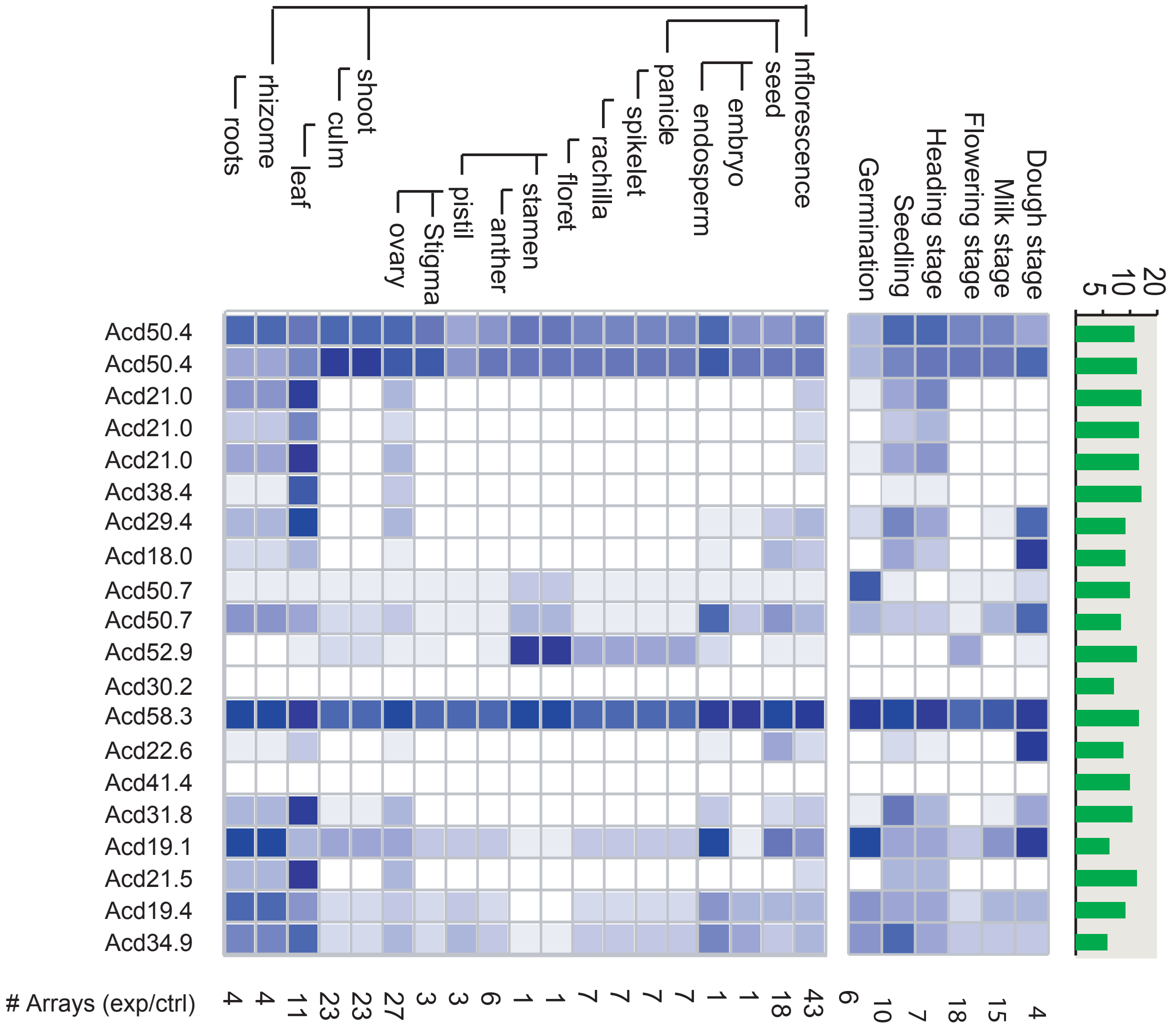

Supplemental figure 3 (A). Microarray based expression profiles of Acd genes of rice during development stages of rice plant. Expression pattern of Acd genes during developmental stages are presented as heat maps in blue /white colors generated using meta-analysis tool at Genevestigator (www.genevestigator.ethz.ch). The colors reflect mean signal values where the darkest color corresponds to stronger expression.

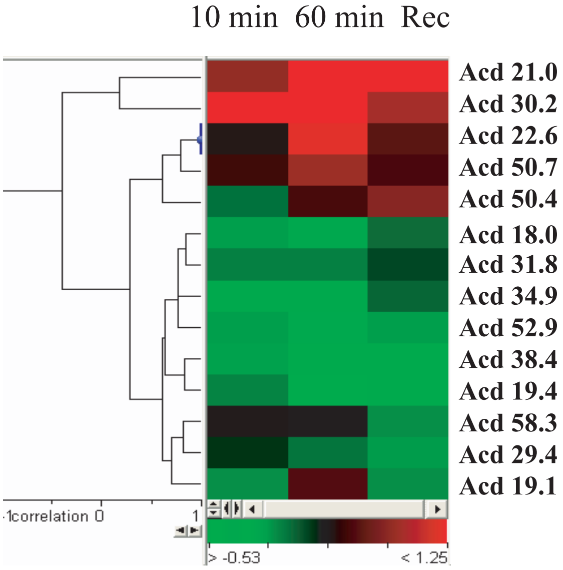

Supplemental figure 3 (B). Microarray based expression profiles of Acd genes of rice under heat stress. Leaves from one month old plants were given stress at 42°C. Samples were harvested after 10 min and 1 h of HT treatment and 30 min recovery following 1 h HT. Control samples were kept at 26°C. The color bar represents log_2 expression values.
