# Supplementary material for: Rice sHsp genes: genomic organization and expression profiling under stress and development
Source: BMC Genomics. 2009 Aug 24;10:393. doi: 10.1186/1471-2164-10-393 (PMC2746236; doi:10.1186/1471-2164-10-393)
Supplement: Additional file 7 — Manual analysis of promoters of sHsp genes. [file 1471-2164-10-393-S7.pdf]

NAME	SITES (5'→3')	START
Hsp17.9A-CI	CTCGAGTTTGTTCTGGACGATTCGGGTCGTGCT	270
Hsp17.4-CI	TACTGGAAGTTTTGCTTCCGGATAGTTCTCGTTTGTTCGTTAAT	348
	GAAGTGGTCTTCTGGATTGTTCTAGGCCTGCTT	302
	AGAGAGCTTGTTGATCTTTCGA	520
Hsp18.0-CI	AAGCAGGCCTAGAACAATCCAGAAGACCACTTC	234
Hsp17.7-CI	AGCTAGAGTAGAAAGTTCCCGAATTACTAAAA	507
	CCACAGGGCCAAAAGTTCTGGAAGGCACTCGATGATTC	250
**Hsp16.9C-CI**	ATGAATACTCTAGAGCAAATTAAGGTGAG	273
	TGGAGCGACGGAAGCGAAGCAGATCGAGTGTGCTG	210
	AGAGAAGTCCAGAGATTTCAGGACAGATTAG	157
**Hsp16.9A-CI**	TCGAGACGCCTCAAGAACGTCCATTGCGCAGCTCC	175
	GAAGCAAAGCCTTTTCTAGAAGGGAGCAGATCAA	240
Hsp16.9B-CI	GAGATGCTTCAAGACCAAGTTCTGAAGAA	152
**Hsp16.6-CVIII**	CTCCAGAACAAGCAGGAAACCTCATCACTCACC	217
	ACTCCTCGACCCTTCACGACGCATCCAA	188
**Hsp17.9B-CIX**	ACGCTGGAGAAGAAGAAGGAATGGCGCCGATCGCC	310
**Hsp23.6-MII**	AATTATGAATCACCGAGAATGTACTCAGAAAAA	583
**Hsp17.8-CXI**	GAAAACGCGAGAAACTACCAGAAAAATCGCGTGTTC	214
	ACACGCGTTCTCGATCATTCTCGCGCGCCACC	97
Hsp23.2-ER	GATCCTTCTCTTTTCTTCCGGTCTGTACAAT	510
	CTCGATCCTTCGGGAAACCTCTCCCGCCG	214
**Hsp22.3-CVI**	AGTGGAAATCTCCAGAAAAAGGAAAAAAAG	194
**Hsp18.8-CV**	TTTAGATTTACATTCGTGAAATAAACCAAA	356

Manual analysis of promoters of sHsp genes.
In addition to MEME based analysis, sHsp genes were analyzed manually. The HSEs which were not identified in promoters by MEME analysis are displayed in the figure. Consensus nucleotides of HSEs are shown in pink font.
